# Differentiation of Epoxide Enantiomers in the Confined Spaces of an Homochiral Cu(II) Metal‐Organic Framework by Kinetic Resolution

**DOI:** 10.1002/chem.202101367

**Published:** 2021-07-09

**Authors:** Juanjo Cabezas‐Giménez, Vanesa Lillo, José Luis Núñez‐Rico, M. Nieves Corella‐Ochoa, Jesús Jover, José Ramón Galán‐Mascarós, Anton Vidal‐Ferran

**Affiliations:** ^1^ Institute of Chemical Research of Catalonia (ICIQ) and the Barcelona Institute of Science and Technology (BIST) Av. Països Catalans 16 43007 Tarragona Spain; ^2^ Departament de Química Física i Inorgànica Universitat Rovira I Virgili (URV) C/Marcel⋅lí Domingo s/n 43007 Tarragona Spain; ^3^ Departament de Química Inorgànica i Orgànica Universitat de Barcelona C/Martí i Franqués 1–11 08028 Barcelona Spain; ^4^ Catalan Institution for Research and Advanced Studies (ICREA) Pg. Lluís Companys 23 08010 Barcelona Spain; ^5^ Institut de Nanociència i Nanotecnologia (IN2UB) Universitat de Barcelona 08028 Barcelona Spain

**Keywords:** amino acids, chirality, epoxides, kinetic resolution, metal-organic framework

## Abstract

**TAMOF‐1**, a homochiral metal‐organic framework (MOF) constructed from an amino acid derivative and Cu(II), was investigated as a heterogeneous catalyst in kinetic resolutions involving the ring opening of styrene oxide with a set of anilines. The branched products generated from the ring opening of styrene oxide with anilines and the unreacted epoxide were obtained with moderately high enantiomeric excesses. The linear product arising from the attack on the non‐benzylic position of styrene oxide underwent a second kinetic resolution by reacting with the epoxide, resulting in an amplification of its final enantiomeric excess and a concomitant formation of an array of isomeric aminodiols. Computational studies confirmed the experimental results, providing a deep understanding of the whole process involving the two successive kinetic resolutions. Furthermore, **TAMOF‐1** activity was conserved after several catalytic cycles. The ring opening of a *meso*‐epoxide with aniline catalyzed by **TAMOF‐1** was also studied and moderate enantioselectivities were obtained.

## Graphical abstract

**The homochiral metal‐organic framework TAMOF‐1** was used as a heterogeneous catalyst in the kinetic resolution of styrene oxide by ring opening reactions with anilines. The linear products underwent a second KR resulting in an amplification of the final stereoselectivity (subtractive Horeau amplification effect). Computational studies rationalized the two successive KR while providing a deep understanding of the process.

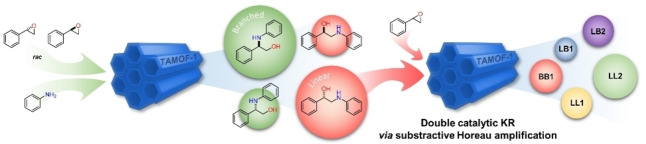

## Introduction

Metal‐organic frameworks (MOFs) are porous coordination polymers composed of organic linkers and metal centers or clusters. With high porosities and thermal stabilities, these materials have captured the attention of many researchers due to their remarkable^[1]^ chemical and physical versatility. They have been used as gas storage^[2]^ and (chiral) separation materials,[^3^, ^4^] sensors,^[5]^ LEDs,^[6]^ drug delivery materials^[7]^ and catalysts,^[8]^ among other applications.

Homochiral MOFs may be obtained from enantiopure precursors. Well‐known enantiopure linkers have been used in the construction of homochiral MOFs, such as BINOL‐ or salen‐based porous materials (BINOL=[1,1′‐binaphthalene]‐2,2′‐diol; salen=2,2′‐((1*E*,1′*E*)‐(ethane‐1,2‐diylbis(azaneylylidene))bis‐(methaneylylidene))diphenol).^[9]^ These MOFs, possessing rigid structures and tunable pores, have been exploited in asymmetric catalysis and chiral separations, two areas of high interest in the pharmaceutical industry. Homochiral MOFs have already been used as enantioselective catalysts for directing the preferential formation of a stereoisomer from a pro‐chiral substrate (for instance, in the cyanosilylation of aldehydes,^[10]^ epoxidation of alkenes,^[11]^ additions to carbonyl groups,^[12]^ carbonyl‐ene^[13]^ and Diels‐Alder reactions,^[14]^ cyclopropanations,^[15]^ aldol reactions,^[16]^ and the ring opening of *meso*‐epoxides with nucleophiles^[17]^). Kinetic resolution is also an interesting tool in enantioselective catalysis for the generation of enantiopure (or highly enantioenriched) products.^[18]^ The success of a catalytic kinetic resolution relies on distinct reaction rates occurring in the presence of a catalyst for the reaction between the two enantiomers of the starting material and an additional reactant/reagent. There are very few examples in the literature of kinetic resolutions of epoxides with homochiral MOFs as catalysts. Moreover, these examples mainly comprise the reaction of oxygen‐containing nucleophiles and epoxides outside the confined spaces of the MOFs.[^19^, ^20^]

**Figure 1:**
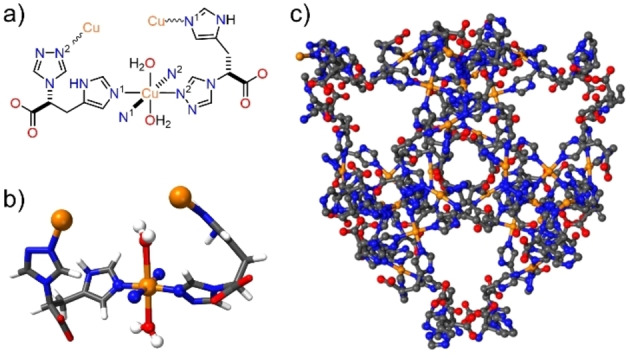
Representation of the Cu_2_L_2_(H_2_O)_2_ building unit: a) schematic, b) ball and stick, and c) packing diagram of 3D **TAMOF‐1** (colour code: C=grey, N=blue, O=red, Cu=orange, H=white; most of the H atoms have been omitted for clarity).

Our research group has recently reported a new homochiral MOF, **TAMOF‐1** (Figure 1), constructed from copper(II) and a linker prepared from natural L‐histidine. The strong metal‐nitrogen coordination bonds of **TAMOF‐1** produce a structurally robust porous material, with a very high permanent porosity (ca. 1200 m^2^ g^−1^) and excellent thermal and chemical stabilities. **TAMOF‐1** has been used as a chiral stationary phase in chromatographic separations for the quantitative separation of racemic mixtures of drugs (for instance, *rac*‐ibuprofen and *rac*‐thalidomide), as well as in HPLC columns for highly efficient analytical separations of racemic mixtures. The 3D intersected channels with a diameter ≤10 Å and the internal binding sites for chiral molecular recognition led to the fabrication of in‐house HPLC columns that outperformed several commercial HPLC columns. The interesting properties of **TAMOF‐1** ^[21]^ (i. e., capability of chiral recognition inside wide channels combined with broad tolerance to solvents) prompted us to study its catalytic activity in kinetic resolutions within confined spaces. Herein, we describe our interesting findings for the kinetic resolution of styrene oxide with a set of anilines in the presence of **TAMOF‐1**. The unreacted epoxide and products generated from the ring opening at the benzylic position were kinetically resolved in an efficient way. Computational studies provided a deep understanding of the whole process, based on the different reactivity of the substrates when confined in the helical channels of **TAMOF‐1**.

## Results and Discussion

### Reaction conditions for TAMOF‐1‐catalyzed ring openings of epoxides with anilines

**Scheme 1:**
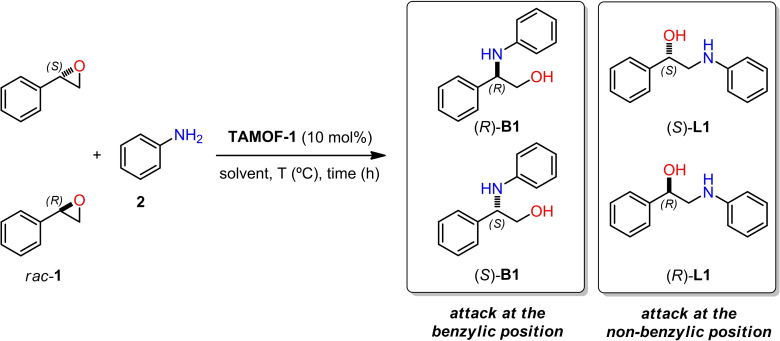
Ring‐opening of *rac*‐**1** with **2** catalyzed by **TAMOF‐1**.

We first explored the resolution of racemic styrene oxide (*rac*‐**1**) with aniline (**2**) to assess the potential of **TAMOF‐1** as a catalyst in the corresponding ring‐opening reaction (Scheme 1). The ring opening of *rac*‐**1** with **2** can generate four different products arising from the nucleophilic attack on the benzylic methine carbon of each enantiomer of the epoxide (branched products, **B1**) or on the non‐benzylic methylene carbon (linear products, **L1**). We used standard screening conditions (10 mol% of catalyst and equimolar amounts of the reagents) in different solvents at different temperatures (Table 1, Supporting Information). Control experiments in the absence of the catalyst revealed that ring opening did not take place to a measurable extent. In the presence of **TAMOF‐1**, the ring‐opening reaction proceeded smoothly, yielding variable amounts of the four possible products depending on the solvent used and the reaction temperature. In all cases, ring opening took place mostly by the nucleophilic attack on the benzylic carbon and involved the formation of **B1**. Conversions of the ring opening with solvents such as toluene, tetrahydrofuran and ethyl acetate were poor, both at low and high temperatures. Consequently, these solvents were not used any further in our studies. Acetonitrile (ACN) was an adequate solvent, with conversions ranging from 26 % to 84 % in the temperature range of 25 °C to 100 °C, respectively. As expected, the enantiomeric excesses (ee's) of the branched and linear products increased by lowering the temperature. The best ee's with ACN were found at rt (36.6 % for (*R*)‐**B1** and 15.2 for (*S*)‐**L1**), although the ee's at 40 °C were quite similar (33.4 % for (*R*)‐**B1** and 7.1 for (*S*)‐**L1**). As the conversion was significantly higher at 40 °C than at rt (53 % vs. 26 %), further studies were performed in ACN at 40 °C, as these reaction conditions provided a good balance between conversion and stereoselectivity.

At this point of the study, the effects of the epoxide/amine ratio were also assessed (Table 1, Supporting Information, entries 17–18). There were no effects on the regioselectivity or enantioselectivity of the final products. Thus, the concentrations indicated above were used in subsequent catalytic studies. Other control experiments were performed to determine whether racemization of the starting materials or products was taking place. These processes were studied by reacting enantiopure (*R*)‐**1** or (*S*)‐**1** separately with aniline (**2**) in the presence of **TAMOF‐1** under the same reaction conditions as those in the catalytic assays. The products obtained were diastereomerically and enantiomerically pure, as expected for an epoxide ring opening process through a stereospecific S_N_2 reaction without any epimerization.

**Scheme 2:**
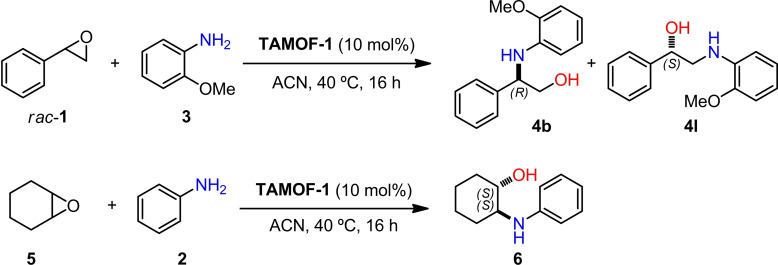
Ring‐opening of *rac*‐**1** and **5** with aniline derivatives **2** and **3** catalyzed by **TAMOF‐1**.

In order to expand the scope of this reaction, 10 mol% of **TAMOF‐1** was used as the catalyst in the reaction of *rac*‐**1** and *o*‐anisidine **3**, with similar results to those reported for aniline being observed (51 % conv.; 60 % ee for (*R*)‐**4 b** and 35 % ee for (*S*)‐**4 l**; see Scheme 2 and Table 2, Supporting Information, for details). In the reaction of the dialkyl‐substituted *meso*‐epoxide **5** and aniline with 10 mol% of **TAMOF‐1** as catalyst, lower reactivity and stereoselectivity were observed than those for the phenyl‐substituted analogue **1** (15 % conversion and 35 % ee in favor of product (1*S*,2*S*)‐**6**; see Scheme 2 and Table 3 in the Supporting Information for details).

### Monitoring of the kinetic resolution

**Figure 2:**
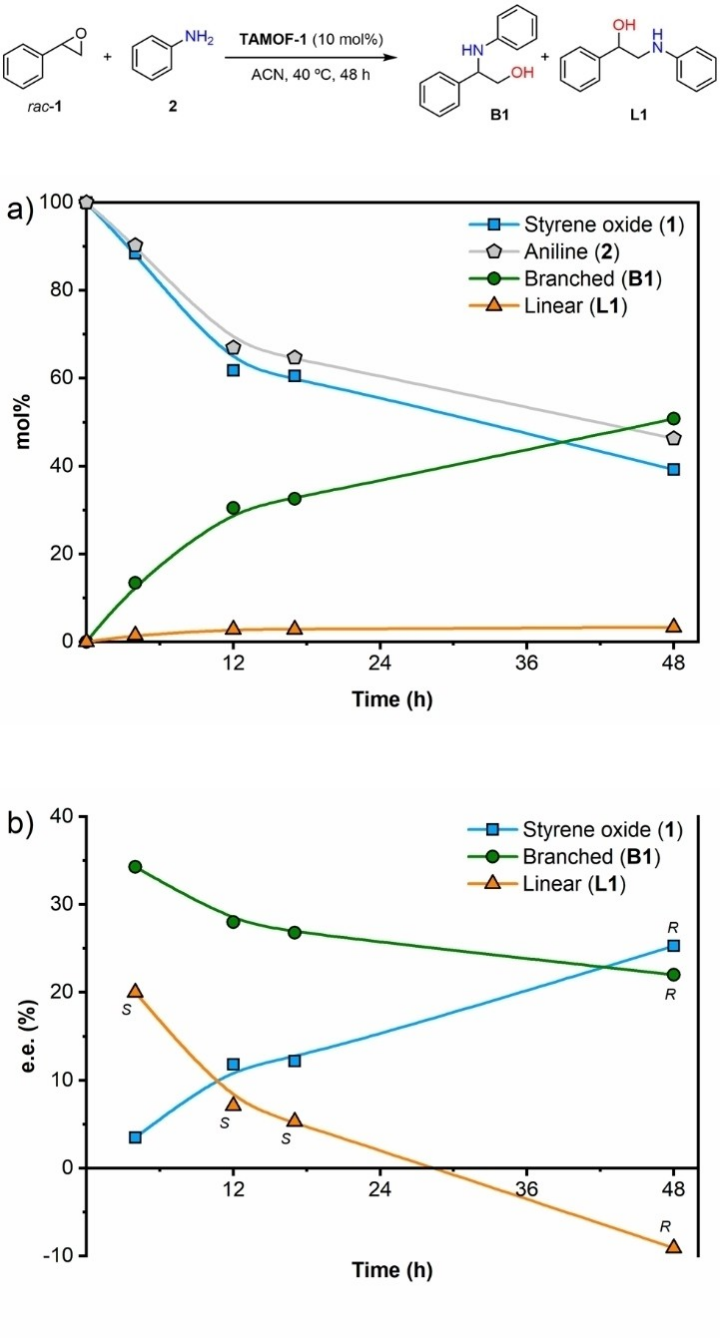
a) Kinetic resolution profile in mol % at 4, 12, 17 and 48 h, using 10 mol % of **TAMOF‐1**. b) Enantiomeric excess of **1**, **B1** and **L1** against time. (Absolute configurations of the products are provided for each compound. The absolute configuration of the product is the same throughout the whole reaction coordinate when the stereodescriptor *R* or *S* is indicated once only). Lines correspond to eye guidelines.

Using the optimized conditions, we studied the kinetic resolution of *rac*‐**1** by measuring conversions and enantiomeric excesses at different reaction times (4, 12, 17 and 48 h). The results are summarized in Figure 2. The concentrations of **1** and **2** decreased rapidly, yielding **B1** as the major product and small amounts of **L1**. Regarding the stereoselectivity of the kinetic resolution, the ee of the unreacted starting material increased with the conversion, whilst the ee of the products dropped as the conversion increased. These trends are in agreement with what is expected in a kinetic resolution.^[18]^ Ring opening of the (*R*)‐enantiomer of **1** with aniline proceeded at a lower rate when using **TAMOF‐1** as the catalyst, with the concentration of (*R*)‐**1** increasing with time. As shown in Figure 2, an ee of 25.3 % in favor of (*R*)‐**1** was recorded after 48 h. As (*S*)‐**1** reacts faster with aniline, the major product formed was the branched product derived from (*S*)‐**1** by the attack of aniline on its benzylic carbon with an inversion of the configuration at the attacked carbon and retention at the other.

**Figure 3:**
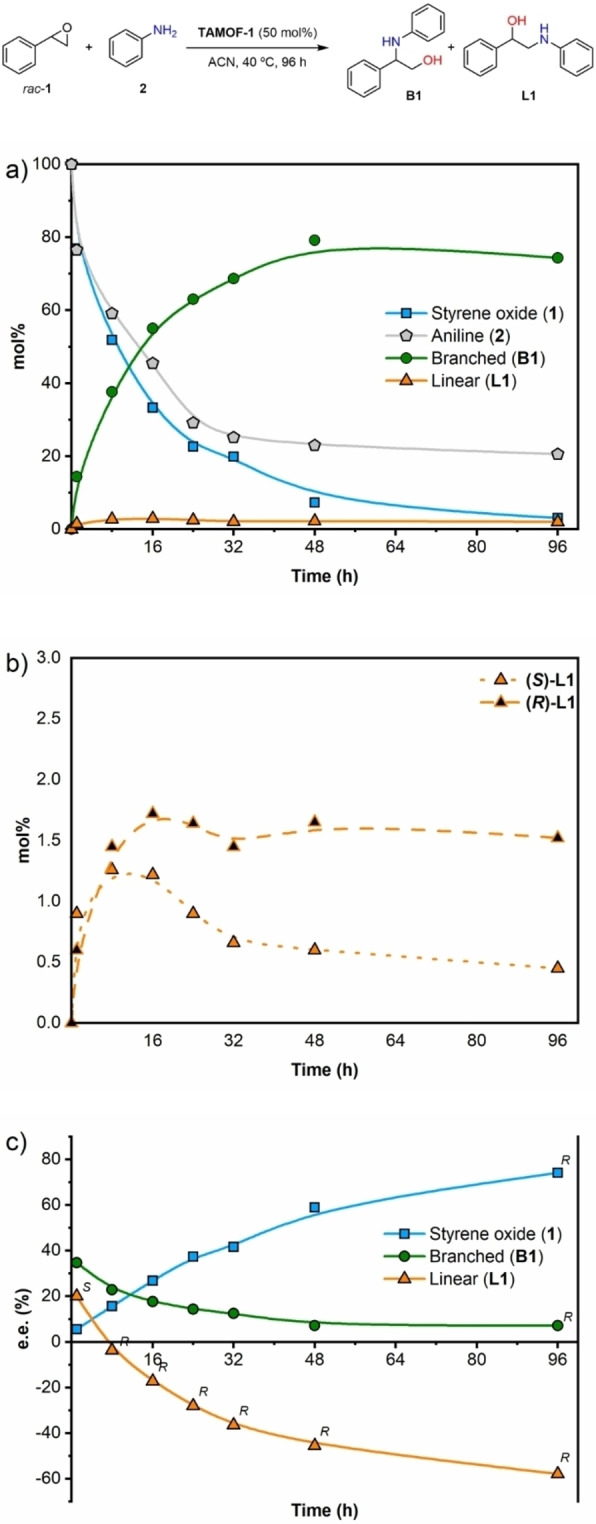
a) Kinetic resolution profile in mol % at 1, 8, 16, 24, 32, 48 and 96 h, using 50 mol% **TAMOF‐1**. b) Kinetic resolution profile of (*R*)‐L**1** and (*S*)‐**L1** in mol %, at same reaction times than (a), using 50 mol% of **TAMOF‐1**. (c) Enantiomeric excesses of **1**, **B1** and **L1** against time. (Absolute configuration of the products are provided for each compound. The absolute configuration of the product is the same throughout the whole reaction coordinate when the stereodescriptor is mentioned only once). Lines correspond to eye guidelines.

Consequently, the branched product (*R*)‐**B1** was mostly formed and its absolute configuration was confirmed by comparing with previous data.^[22]^ The ee for this compound after four hours was 34.3 %. The product arising from the attack of aniline on the non‐benzylic carbon of the two enantiomers of the starting epoxide (**L1**) was also observed in the reaction mixture. However, **L1** was formed in lower amounts compared to **B1**. A comparison of the yields of **B1** and **L1** as a function of time (Figure 2a) shows that the yield of **L1** was lower than that for **B1** throughout the whole reaction coordinate. The stereoselectivity of **L1** after four hours was slightly lower than that for **B1** (ee of 20.0 % for (*S*)‐**L1** and 34.3 % for (*R*)‐**B1**). This indicates a slightly lower degree of stereodifferentiation of the two enantiomers of the starting material by **TAMOF‐1** for the non‐benzylic position. Surprisingly, an inversion of the configuration of the product **L1** was observed at long reaction times (formation of (*R*)‐**L1** with an extended ee of 9.1 %). This finding was not consistent with a simple kinetic resolution. To further understand the stereochemical outcome of this reaction, we performed analogous experiments with increased **TAMOF‐1** amounts (from 10 mol% to 50 mol%) and extended reaction times. The results of this study are summarized in Figure 3. With the new reaction conditions, the conversion of the ring opening increased up to 97 % after 96 h.

**Figure 4:**
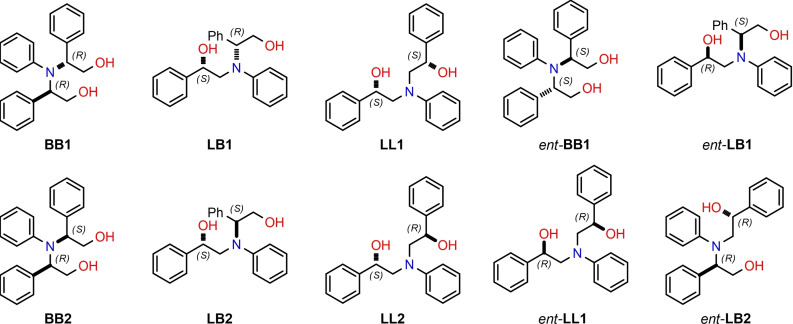
Isomeric aminodiols derived from the reaction between two units of *rac*‐**1** and one unit of **2** (all of them show the molecular ion [M+H]^+^ at m/z=334.4, as expected for C_22_H_24_NO_2_^+^).

Despite both the ee of the unreacted starting material and the stereoselectivity pattern of **B1** as a function of time being in agreement with a standard kinetic resolution, the final configuration of **L1** using 50 mol% of **TAMOF‐1** was again opposite to the one expected for ring opening at the non‐benzylic position of the fastest reacting enantiomer of **1** with **2**, as observed with 10 mol% of **TAMOF‐1**. Analyses in an HPLC system equipped with an atmospheric pressure chemical ionization detector in the positive mode (APCI^+^) of crude reaction mixtures identified unexpected reaction products, which were consistent with adducts formed from one molecule of aniline and two molecules of styrene oxide. These findings suggested that a second ring‐opening reaction was taking place, since the products arising from the first ring‐opening (i. e., **B1** and **L1**) still had the ability to attack a second molecule of styrene oxide to form dialkyl aryl tertiary aminodiols. It should be noted that the ring opening of a second unit of a racemic epoxide, at the benzylic and non‐benzylic positions, by amino alcohols (*R*)‐**B1**, (*S*)‐**B1**, (*R*)‐**L1** or (*S*)‐**L1** may generate up to 10 diastereoisomeric aminodiols, whose structures are shown in Figure 4. Interestingly, five peaks with [M+H]^+^ ions that were consistent with the suggested aminodiol structures were observed. In particular, **LB1, LL1, LB2** and **LL2** were identified in the reaction mixtures by comparative HPLC analyses of the crude reaction mixtures and pure samples of these compounds.^[23]^

In addition, the aminodiol **BB1** arising from the ring opening of the fastest reacting enantiomer (*S*)‐**1** by the most abundant amino alcohol (*R*)‐**B1** was unequivocally identified in the reaction mixture by comparative HPLC analysis.^[23]^

### Matched double catalytic kinetic resolutions via subtractive Horeau amplification

**Figure 5:**
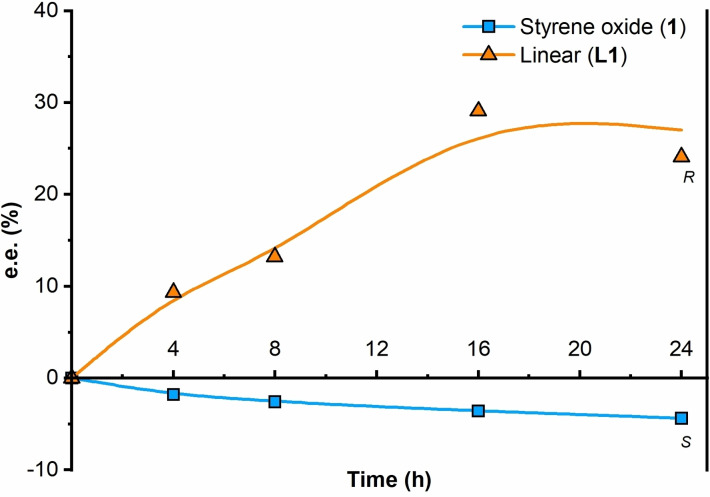
Enantiomeric excess profile of the second kinetic resolution. The kinetic resolution takes place with the **L** isomer, where (*R*)‐**L1** reacts faster than (*S*)‐**L1**. (The absolute configuration of the product is the same throughout the whole reaction coordinate and the stereodescriptor is indicated on the graphic line). Lines correspond to eye guidelines.

**Scheme 3:**
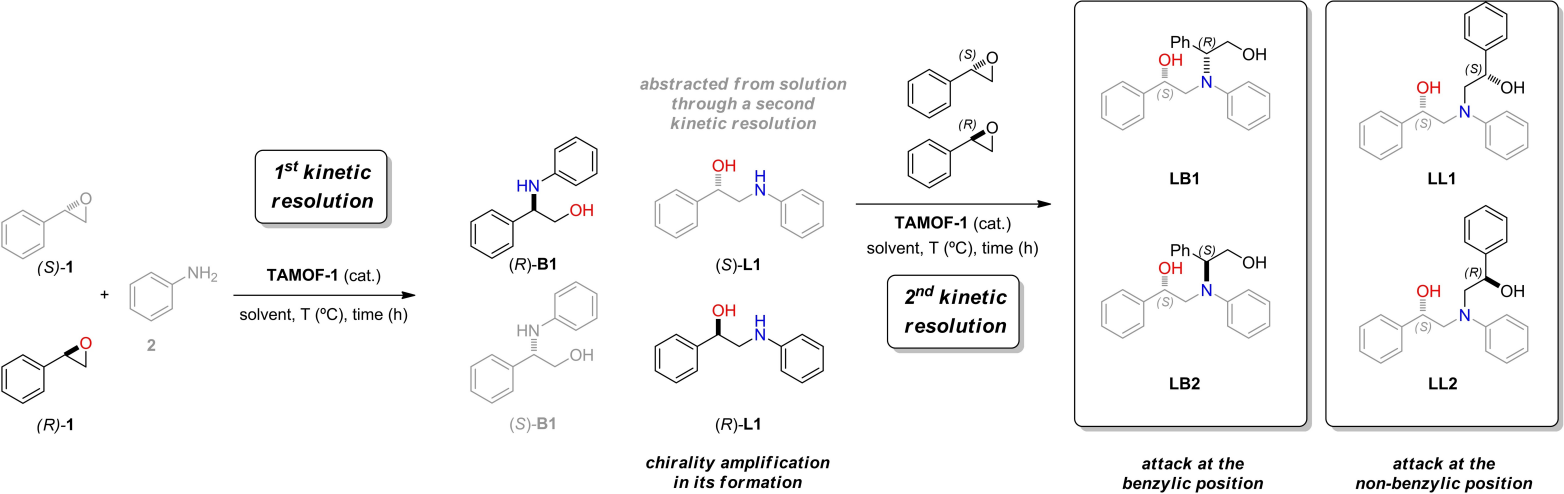
General scheme of the whole kinetic resolution process.

Intrigued by the origin of the inversion of the configuration of **L1** at high conversions, we looked closely at the concentration profile of both enantiomers of **L1** vs. time (see Figure 3b). At short reaction times, there was an excess of (*S*)‐**L1** with respect to (*R*)‐**L1**. As previously indicated, formation of (*S*)‐**L1** in the presence of **TAMOF‐1** was slightly preferred in the reaction between *rac*‐**1** and **2**. However, (*S*)‐**L1** reacted faster than (*R*)‐**L1** with styrene oxide to form the corresponding aminodiol. This can be seen in Figure 3b, where the concentration of (*S*)‐**L1** decreased with time (dotted curve in Figure 3b), whilst that of (*R*)‐**L1** reached a plateau and remained quite constant (dashed line in Figure 3b). As (*S*)‐**L1** was abstracted from the reaction media forming **LL1**, the whole process led to the formation of **L1** with an increased ee of 57.9 % in favor of the (*R*)‐enantiomer (orange curve in Figure 3c). These observations were confirmed by performing the kinetic resolution of *rac*‐**L1** with *rac*‐**1** in the presence of **TAMOF‐1** (Figure 5). As shown in this figure, the kinetic resolution of *rac*‐**L1** led to (*R*)‐**L1**, leaving (*S*)‐**1** as the unreacted enantiomer of the starting epoxide. It should be noted that (*R*)‐**1** is the fastest reacting enantiomer of the epoxide with the amino alcohol (*S*)‐**L1**, whilst the other enantiomer of styrene oxide is the fastest reacting enantiomer with aniline. It is also interesting to note the chiral amplification effect observed in the formation of (*R*)‐**L1** (from an ee of 20.0 % with one kinetic resolution to 57.9 % after two kinetic resolutions). The most abundant enantiomer of the linear product formed after one kinetic resolution (i. e., (*S*)‐**L1**) reacted faster through a second kinetic resolution process than (*R*)‐**L1**, leading to an accumulation of the latter (by abstraction of the former). Overall, through two matched kinetic resolutions, the stereoselectivity of the formation of (*R*)‐**L1** is enhanced. These results constitute an interesting example of subtractive Horeau amplification within the pores of **TAMOF‐1** (see Scheme 3 for a summary of the processes involved).^[24]^

To demonstrate that kinetic resolutions were occurring inside the pores of **TAMOF‐1**, reactants with more demanding steric profiles were used (i. e., racemic *trans*‐2,3‐diphenyloxirane instead of its mono‐substituted analogue *rac*‐**1** and *tert‐*butyl‐amine instead of aniline). Interestingly, kinetic resolution of *trans*‐2,3‐diphenyloxirane and aniline under identical reaction conditions to those used for its monophenyl‐substituted analogue *rac*‐**1** did not take place at any measurable extent. In an analogous manner, the kinetic resolution of styrene oxide with *tert*‐butylamine did not proceed either. These results confirmed that kinetic resolution processes take place inside the pores of **TAMOF‐1**.

### Reaction mechanism

DFT calculations have been employed to investigate the reaction pathways leading to the experimentally observed amino alcohol and aminodiol products in both kinetic resolutions. The computed transition state energies, directly related to the new C–N bonds formed, constituted the key point for each pathway and were able to describe the preferential reactivity of the catalytic system.

**Figure 6:**
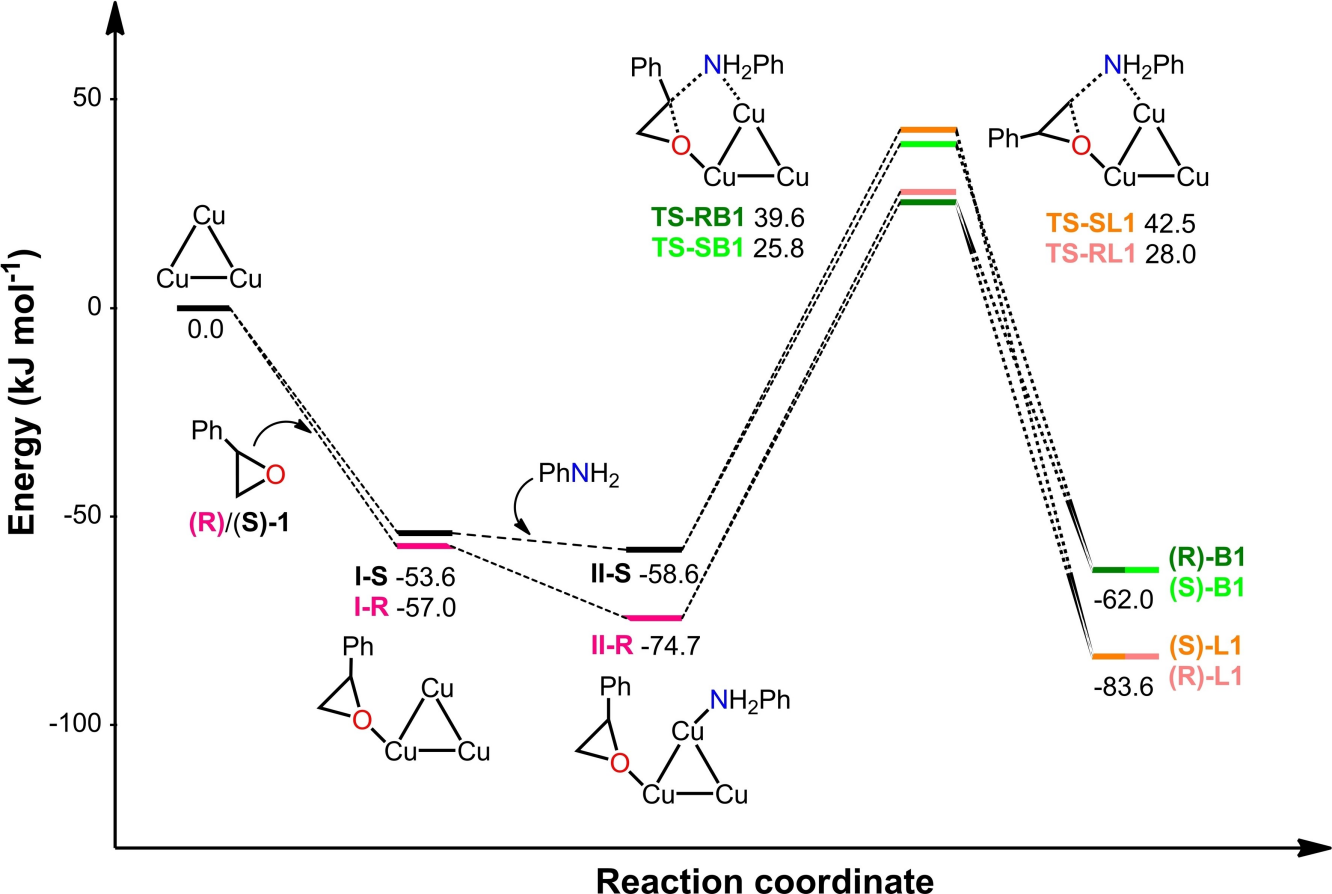
Computed energy profiles (in kJ mol^−1^) for the **TAMOF‐1**‐catalyzed enantioselective ring opening of (*S*)‐**1** and (*R*)‐**1** styrene oxide by aniline.

The reaction mechanism for the **TAMOF‐1**‐catalyzed ring opening of both (*S*)‐**1** and (*R*)‐**1** styrene oxides by aniline (**2**) (Figure 6) to produce (*R*)/(*S*)‐**L1** or (*R*)/(*S*)‐**B1** has been explored. The reaction starts by the coordination of the epoxide **1** to the free axial position of one of the copper atoms in the **TAMOF‐1** structure; this process is exothermic for both styrene oxides by more than 50 kJ mol^−1^. In intermediates **I‐R** and **I‐S**, the distance between the oxygen and the copper is around 2.4 Å. In addition, other weak contacts contribute to the stabilization of these structures (e. g., the hydrogen atoms of the epoxide functionality point to the vacant axial position of the other two copper centers at distances ranging between 2.6 and 2.9 Å).

The epoxide molecules are not strongly activated upon coordination to the copper, as their C−O distances remain practically the same in the free and copper‐bound styrene oxides. Subsequent aniline **2** attachment to one of the remaining two copper atoms leads to the formation of intermediates **II‐R**/**II‐S**. The Cu−N distance in these complexes is around 2.35 Å and, although one of the Cu−H_epox_ contacts is lost, the other one is maintained, helping to stabilize these intermediates. This coordination process is also exothermic, and the associated energy gain varies depending on which enantiomer of styrene oxide is already present within **TAMOF‐1**. Our results indicate that the binding of aniline is ca. 12 kJ mol^−1^ stronger in the presence of (*R*)‐styrene oxide, indicating that the MOF differentiation for both styrene oxide enantiomers takes place in the presence of aniline. After the addition of aniline, the whole cavity is practically full and both substrates lie quite close to each other; additional contacts, shorter than 2.6 Å, are established between the styrene oxide and aniline in both **II‐R** and **II‐S**.

Nucleophilic attack of **2** onto the epoxide takes place producing either the linear or the branched product. The energy barriers leading to the latter are lower for both styrene oxides by 2.2 and 2.8 kJ mol^−1^ for the (*R*)‐**B1**/(*S*)‐**L1** and (*S*)‐**B1**/(*R*)‐**L1** pairs, respectively. Although these energy differences are not very large, they are enough to favor the formation of one regioisomer over the other in agreement with the experimental observations, which state that the branched products (**B1**) are favored. The energy barrier heights range between 98.2 and 102.7 kJ mol^−1^, indicating that the reaction should not be very fast, as observed experimentally. It should be noted that the S_N_2 barriers for the reaction of (*S*)‐**1** with aniline are lower than for its (*R*)‐**1** counterpart: 98.2 and 100.5 kJ mol^−1^ for the branched products, and 101.1 and 102.7 kJ mol^−1^ for the linear products. These differences in barrier heights are responsible for the observed enantioselectivity, with products (*R*)‐**B1** and (*S*)‐**L1** being preferably formed. The intramolecular proton transfer steps to transform the zwitterionic amino alcohols into **B1** and **L1** have not been studied, since they are expected to be fast processes not determining the reaction outcome. The release of the final product is exothermic in all cases, except for (*S*)‐**B1**. In this case, this product should be easily displaced by an incoming styrene oxide or aniline molecules, which would enable the catalytic process to proceed smoothly. As observed, **TAMOF‐1** favors the formation of the branched products **B1** over their –more stable– linear counterparts **L1**, as expected for a transformation that takes place under kinetic control.

**Figure 7:**
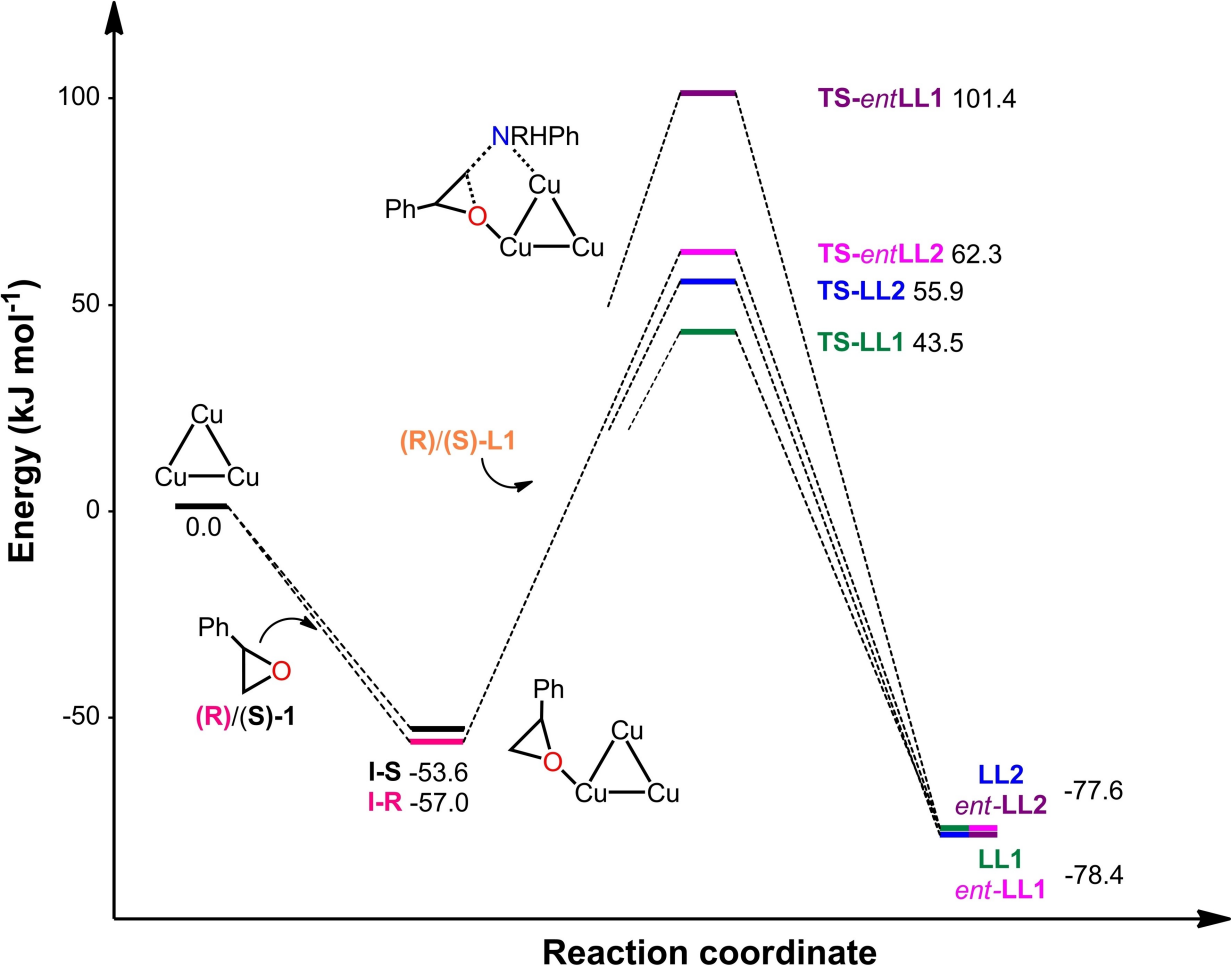
Computed energy profiles (in kJ mol‐1) for the TAMOF‐1‐catalyzed enantioselective ring opening of (*S*)‐**1** and (*R*)‐**1** styrene oxides with (*S*)‐**L1** and (*R*)‐**L1** aminoalcohols.

**Figure 8:**
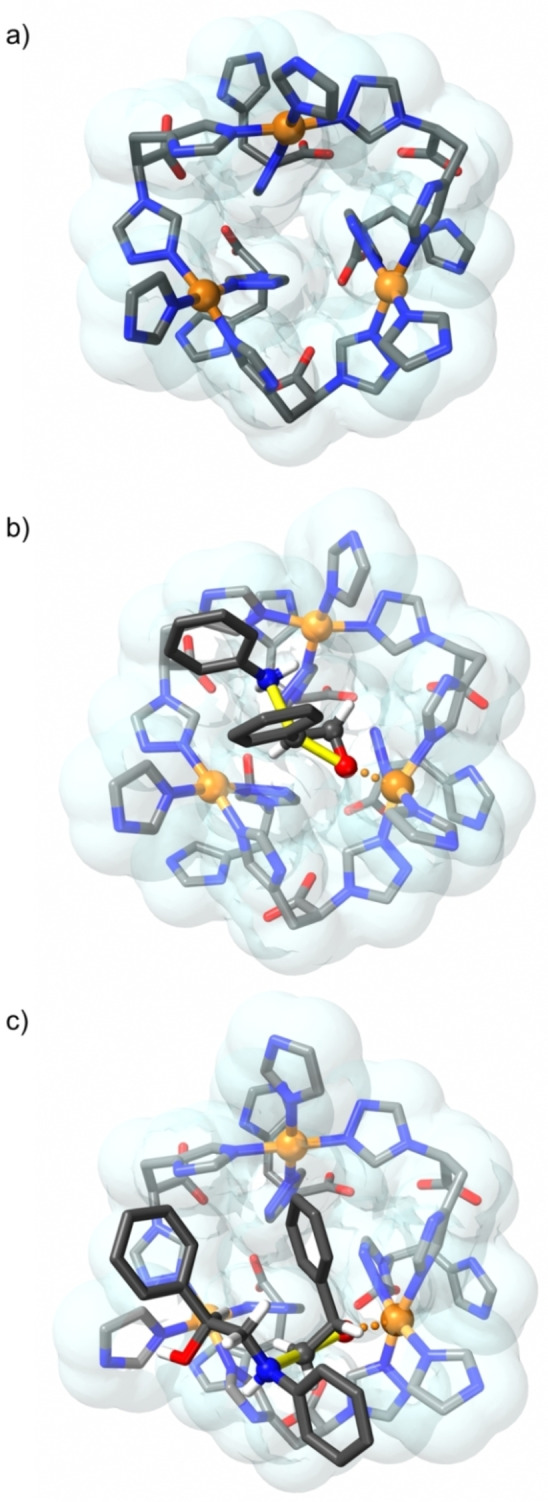
a) Catalytic site of **TAMOF‐1**. b) Catalytic site for the preferred TS of the first ring opening. c) Catalytic centre for the preferred TS of the second ring opening. Bonds being broken/formed are indicated in yellow.

The transition states for the nucleophilic attack of aniline onto *R*‐**1** and *S*‐**1** have also been computed without **TAMOF‐1**. In these cases, the energy barriers leading to the branched and linear products are as high as 155.3 and 148.9 kJ mol^−1^, respectively, around 50 kJ mol^−1^ higher than those computed within **TAMOF‐1**. Interestingly, the uncatalyzed reaction favors the formation of the linear products, which are in all cases the preferred thermodynamic products (see Figure 7 and Figure 8).

These results indicate that the copper atoms in **TAMOF‐1** cooperatively mediate the formation of the products in the first kinetic resolution, probably by bringing styrene oxide and aniline close together inside the **TAMOF‐1** cavities. This effect has been already observed in reactions with supramolecular catalysts such as cucurbiturils, where the encapsulation of the reactants largely reduces the entropic cost of bringing reagents together and enhances reaction rates.^[25]^ In addition, the coordination of one aniline and one styrene oxide molecules within **TAMOF‐1** has a positive accumulation effect on the reaction rate; the formation of species **II‐S** and **II‐R** is energetically favored over other possible non‐productive bimolecular arrangements containing either one aniline and one acetonitrile, one styrene oxide and one acetonitrile, and two aniline molecules.^[26]^ Therefore, **TAMOF‐1** has two different functions in this process: first, it lowers the reaction barrier leading to the formation of all products, and second, allows for the preferential formation of the kinetic products over their thermodynamic counterparts, in contrast to the uncatalyzed process. The observed final configuration of **L1** enantiomers at long reaction times, producing a second kinetic resolution of aminodiols, has also been computed. In particular, we have studied the **TAMOF‐1**‐catalyzed reaction between (*R*)/(*S*)‐**L1** intermediates and (*R*)/(*S*)‐**1** to deliver the linear products **LL1**, **LL2**, and their enantiomers, which constitute the most abundant products at this stage (Figure 7). The second kinetic resolution reaction starts by coordination of styrene oxide to one of the copper atoms in **TAMOF‐1** with this process being exactly the same than that found in the first kinetic resolution. The reaction should subsequently proceed by coordination of **L1** products to **TAMOF‐1**. The computed transition states indicate that the fastest reaction is that between (*S*)‐**L1** and (*R*)‐**1** to produce **LL1**, as experimentally observed (see Figure 7 and Figure 8). This reaction has a barrier of 100.5 kJ mol^−1^; in contrast, the reaction between (*S*)‐**L1** and (*S*)‐**1** to produce **LL2** has a larger barrier of 112.9 kJ mol^−1^, confirming the preference of (*S*)‐**L1** to engage with (*R*)‐**1**.

These results indicate that, under experimental conditions, (*S*)‐**L1** would engage faster in a second C−N bond formation process, hence producing the accumulation of (*R*)‐**L1** and the reversal of the expected enantioselectivity for these amino alcohol intermediates at long reaction times.

The energy barriers for the formation of **LL1** and **LL2** from (*S*)‐**L1** and (*R*)/(*S*)‐**1** have also been computed in the absence of **TAMOF‐1**. The reaction of (*S*)‐**L1** with (*R*)‐**1** and (*S*)‐**1** produces high barriers of 150.4 and 161.4 kJ mol^−1^, respectively. In this case, the reactivity order with and without **TAMOF‐1** is not altered but it can be observed that the presence of the MOF catalyst drastically reduces the energy barriers for most of the reaction pathways.

### Recyclability of TAMOF‐1

**Figure 9:**
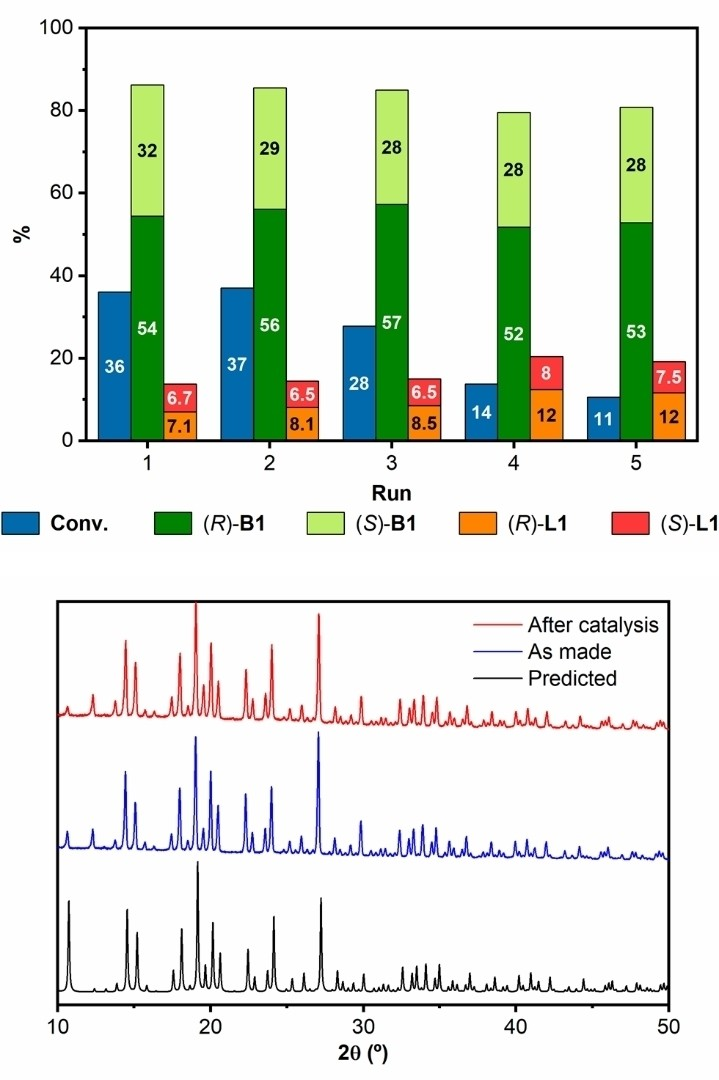
(Top) Conversion and enantiomeric ratio (indicated in the bar graphs) of **B1**, **B2**, **L1** and **L2** after recycling **TAMOF‐1** five times. Reaction conditions: 0.5 mmol of *rac*‐**1**, 0.5 mmol of **2**, **TAMOF‐1** (10 mol%) and 2 mL of ACN at 40 °C for 16 h. (Bottom) PXRD comparison of **TAMOF‐1** after one catalytic cycle (red pattern), as made (blue pattern) and simulated from its crystal structure (black pattern).

Recyclability is another important feature for heterogeneous catalysts. To assess the performance of **TAMOF‐1** as a recoverable and reusable catalyst, a series of reaction cycles were performed. After each run (ACN, 40 °C, 16 h), the catalyst was collected through filtration, dried in the oven at 40 °C and reused in the next run of reactions under the same conditions. There was no loss of reactivity and enantioselectivity in the first two reaction cycles, whilst catalytic activity in terms of conversion was affected after the third run (Figure 9, top), attributed to a loss of catalyst due to handling between cycles.

Regioselectivity and stereoselectivity remained constant throughout the five consecutive reaction cycles. Moreover, the crystallinity of the used catalyst after one reaction cycle remained unaltered as compared to that of freshly prepared catalyst (Figure 9, bottom).

## Conclusion

In conclusion, **TAMOF‐1** is a recyclable catalyst for the efficient kinetic resolution of *rac*‐styrene oxide (**1**) with a set of anilines, yielding secondary amino alcohols. The linear products arising from the attack on the non‐benzylic position underwent a second kinetic resolution, resulting in an amplification of its final enantiomeric excess and a concomitant formation of an array of isomeric aminodiols (subtractive Horeau amplification effect). The ring opening of a *meso*‐epoxide with aniline catalyzed by **TAMOF‐1** was also studied and moderate enantioselectivities were obtained. Computational studies on the whole process have provided deep insight into the ring opening steps and stereochemical outcome of the sequential kinetic resolution processes. These studies have revealed that **TAMOF‐1** lowered the reaction barriers of all ring opening processes taking place inside channels. The calculated energy barriers of the reactions leading to the ring opening products were in complete agreement with the experimental observations: preferential formation of (*R*)‐**B1** in the first kinetic resolution and preferential consumption of (*S*)‐**L1** in the second kinetic resolution (with concomitant amplification of the chirality for the unreacted (*R*)‐**L1** amino alcohol). The regioselectivity and enantioselectivity of **TAMOF‐1** remained constant throughout five consecutive reaction cycles.

## Experimental Section

### Materials and general

All syntheses were carried out on chemicals as purchased from commercial sources, unless otherwise indicated. Air and moisture sensitive manipulations or reactions were run under inert atmosphere using anhydrous solvents and Schlenk techniques. All solvents were either purchased commercially anhydrous or dried by using a Solvent Purification System (SPS). Silica gel 60 (230–400 mesh) was used for column chromatography. NMR spectra were recorded in CDCl_3_ as solvent on a Bruker Advance 300, 400 or 500 MHz Ultrashield spectrometers. ^1^H and ^13^C NMR chemical shifts are quoted in ppm relative to residual solvent peaks. Data for ^1^H NMR are given as follows: chemical shift, multiplicity (s=singlet, d=doublet, t=triplet, m=multiplet, dd=doublet of doublets, tt=triplet of triplets), coupling constants and intensities. High‐resolution mass spectra (HRMS) were recorded by using ESI as ionization method in positive mode. IR spectra were recorded using Attenuated Total Reflection (ATR) spectroscopy. Specific optical rotation ([α]_λ_^T^) was recorded using a Jasco P1030 Polarimeter with a wavelength of 589 nm (sodium, D line) at 25 °C in the corresponding solvent, in a 10 cm cell, unless otherwise indicated. Crude mixtures of the kinetic resolution or *rac*‐**1** were analyzed by HPLC chromatography with an Agilent 1200 Series system equipped with a Chiralpak® IC column (4.6 x 250 mm) and a diode array detector, at a flow rate of 1 mL/min and a gradient solvent system of Hex/IPA 99 : 1 to 95 : 5 [(min, %IPA): (0, 1); (7, 1); (14, 5); (40, 5)], unless otherwise indicated.

### General procedure for the kinetic resolution of *rac*‐1

Prior to start the reaction, **TAMOF‐1** (10 or 50 mol%) was activated at 130 °C *in vacuo* for at least 3 h in a Schlenk flask. The Schlenk flask was filled with nitrogen and left to cool at rt, then, a solution of *rac*‐**1** (1 equiv.) and the corresponding amine (1 equiv.) in ACN (0.25 M) was added to the Schlenk flask and the reaction mixture was stirred under nitrogen atmosphere at the desired temperature for the required time. The reaction mixture was left to cool at rt, then the suspension was filtered using a syringe filter with PTFE membrane (0.2 μm) and the filter was washed three times with ACN. Crude products of catalytic reactions were analyzed in an Agilent 1200 series HPLC system equipped with diode array detector and quantified using external standards.

**(*R*)‐B1**: ^**1**^ **H NMR** (400 MHz, CDCl_3_) δ 7.41–7.31 (m, 4H), 7.31–7.24 (m, 1H), 7.16–7.05 (m, 2H), 6.75–6.65 (m, 1H), 6.63–6.55 (m, 2H), 4.51 (dd, *J*=7.0, 4.2 Hz, 1H), 3.95 (dd, *J*=11.1, 4.2 Hz, 1H), 3.76 (dd, *J*=11.1, 7.0 Hz, 1H). ^**13**^ **C{^1^H} NMR** (101 MHz, CDCl_3_) δ 147.37 (C), 140.25 (C), 129.30 (CH), 128.99 (CH), 127.77 (CH), 126.87 (CH), 118.05 (CH), 114.00 (CH), 67.53 (CH), 60.02 (CH_2_). **HRMS‐ESI** (*m/z*): [M+H]^+^ calcd for C_14_H_16_NO, 214.1226; found, 214.1219. **[α]^25^**_**D**_=–21.9 (c=0.50, CHCl_3_), lit. (*S*)‐B1=+27.5 (c=0.50, CHCl_3_);^[27]^ **IR** (neat, cm^−1^) ν 3396, 3053, 3025, 2927, 2873, 1600, 1502, 1451, 1430, 1353, 1315, 1264, 1180, 1064, 1027, 749, 693.

### Computational details

The periodic structure of **TAMOF‐1** was truncated when carrying out the computational studies. The trinuclear copper cavity present in the MOF was preserved as such; the metal centers remain connected through 6 *S*‐HTA ligands and the coordination environment for each copper atom is completed by a truncated *S*‐HTA, which has been modeled as a 3‐(1*H*‐imidazol) ligand. This truncated structure is charge neutral and accounts for 168 atoms. All the calculations have been carried out using the Gaussian09^[28]^ software with the unrestricted formalism of the dispersion‐corrected B3LYP(D3)^[29]^ functional and the Ahlrichs def2svp basis set^[30]^ for all atoms. The geometries have been optimized in acetonitrile using the PCM method^[31]^ and employing the SMD solvation model.^[32]^ In all cases the spin multiplicity was kept as a quartet, corresponding to a situation where the three copper(II) atoms do not interact with each other. The nature of the stationary points along the reaction coordinate has been confirmed by vibrational analysis; the number of imaginary frequencies for minima and transition states is zero or none, respectively. Unless otherwise stated, all the reported energy values correspond to relative electronic energy differences corrected to include the translational entropy of the small molecules (aniline, styrene oxide, aminoalcohols, etc.) in THF at 40 °C. In the case of the uncatalyzed transition states, i. e. those where **TAMOF‐1** is not involved, computed Gibbs energies at 40 °C are provided. All the transition states were sought using the linear transit scan method, which consists of a series of optimizations keeping the reaction coordinate of interest fixed at different values. In this case this process corresponds to scanning along the C–N bond that leads to the formation of the four possible enantiomers. When using this method, the structure with the highest energy is considered to be the transition state. All the relevant structures are published in the ioChem‐BD database and can be retrieved in the following link: https://doi.org/10.19061/iochem‐bd‐1‐177.

## Conflict of interest

The authors declare the following competing financial interests: M.N.C.O., V.L. and J.R.G.M. are listed as inventors on the European patent application EP16382480.8, filed by ICIQ and ICREA (priority date: 21/10/2016), which protects the chemical structure of **TAMOF‐1** and its derivatives and analogues, along with their applications, including but not limited to their use in kinetic resolutions. This patent has been licensed to Orchestra Scientific S.L., a spin‐off company founded by J.R.G.M., ICIQ and ICREA.

## Supporting information

As a service to our authors and readers, this journal provides supporting information supplied by the authors. Such materials are peer reviewed and may be re‐organized for online delivery, but are not copy‐edited or typeset. Technical support issues arising from supporting information (other than missing files) should be addressed to the authors.

Supporting Information
