# Supplementary material for: Differentiation of Epoxide Enantiomers in the Confined Spaces of an Homochiral Cu(II) Metal‐Organic Framework by Kinetic Resolution
Source: Chemistry. 2021 Jul 9;27(68):16956–65. doi: 10.1002/chem.202101367 (PMC9291124; doi:10.1002/chem.202101367)
Supplement: Supplementary file 1 — Supporting Information [file CHEM-27-16956-s001.pdf]

Chemistry–A European Journal

Supporting Information

Differentiation of Epoxide Enantiomers in the Confined Spaces of an Homochiral Cu(II) Metal-Organic Framework by Kinetic Resolution

Juanjo Cabezas-Giménez, Vanesa Lillo, José Luis Núñez-Rico, M. Nieves Corella-Ochoa, Jesús Jover,* José Ramón Galán-Mascarós,* and Anton Vidal-Ferran*

Contents

Experimental section 1

Synthesis of (*E*)-*N'*-((*E*)-(Dimethylamino)methylene)-*N*,*N*-dimethylformo-hydrazonamide 1

Synthesis of (*S)*-3-(1*H*-Imidazol-5-yl)-2-(4*H*-1,2,4-triazol-4-yl)propanoic acid (*S*-HTA) 1

Synthesis of $[Cu(H_2O)_2(S\text{-}TA)_2]\cdot 6H_2O$ (TAMOF-1) 2

Screening conditions and results for the kinetic resolution of *rac*-1 and aniline (2) 2

Ring-opening of *rac*-1 with *o*-aniside (3) 3

Ring-opening of *rac*-cyclohexene oxide (5) with aniline (2) 3

Synthesis of products (*R*)-B1, (*S*)-L1, BB1, LB1, LL1[5] 4

Synthesis of products LB2 and LL2 4

Synthesis of *rac*-4b and *rac*-4l[6] 5

Synthesis of (*S*)-4b and (*R*)-4l[7] 5

Synthesis of *rac-trans*-6[8] 6

Results of the kinetic resolutions 6

Characterization of amino alcohols and aminodiols 8

(*R*)-B1 **8**

(*S*)-L1 **8**

BB1 **8**

LB1 **9**

LL1 **9**

LB2 **9**

LL2 **10**

(*S*)-4b **10**

(*R*)-4l **10**

***rac-trans*-6** **10**

NMR spectra 12

HPLC and SFC chromatograms 22

References 27

Experimental section

Synthesis of (*E*)-*N'*-((*E*)-(Dimethylamino)methylene)-*N*,*N*-dimethylformo-hydrazonamide (known product[1])

Part I: 150 mL of ice-cooled dimethylformamide (DMF) were placed in a 250 mL three-neck round-bottom flask (RBF). Afterward, 28.6 mL of $SOCl_2$ (0.39 mol) were slowly added under stirring with a syringe (dropwise). The reaction mixture was stirred for 24 h. After this time, the RBF was introduced in an ice-bath and, once the mixture was cold, a solution of 5 mL of $NH_2NH_2{\cdot}H_2O$ (0.1 mol) dissolved in 15 mL of DMF was slowly added (dropwise and carefully; CAUTION: very exothermic reaction). The reaction mixture was stirred at room temperature for 48 h. The precipitate was then filtered and washed with DMF (20 × 3 mL), Et_2O (10 × 3 mL) and dried in the air to yield 20.5 g (0.095 mol, 95% yield) of **I**.

Part II: 20.5 g of **1** (0.095 mol) were weighed and dissolved in 100 mL of H_2O. In a different beaker, 10.6 g of Na_2CO_3 (0.1 mol) were dissolved in 100 mL of H_2O. Both water solutions were then mixed (the Na_2CO_3 on top of the azine solution), and the desired product **II** was purified by continuous extraction with 750 mL of Et_2O for 2 days. The organic layer was concentrated *in vacuo*, and 12.95 g of 2 (0.09 mol, 96% yield) were obtained as an orange solid, which was used without any further purification.

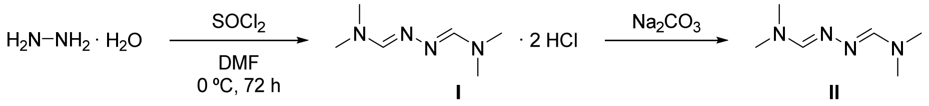

Scheme ESI1. Synthesis of **I** and **II**

Synthesis of (*S)*-3-(1*H*-Imidazol-5-yl)-2-(4*H*-1,2,4-triazol-4-yl)propanoic acid (*S*-HTA) (known product[2])

Compound **2** (6.3 g, 0.044 mol) and L-histidine (3.1 g, 0.02 mol) were mixed together in 150 mL of EtOH. The mixture was heated at reflux under stirring for 48 h. Unreacted L-histidine was filtered off from the reaction mixture, and the solvent was removed under reduced pressure to afford an orange gel that was washed with EtOH to yield a white precipitate of ***S*-HTA**, that was collected by filtration, washed with EtOH (15 × 3 mL), Et_2O (15 × 3 mL) and dried in the air. Yield: 3.5 g (0.017 mol, 85%). Anal. Calcd for $C_8H_9N_5O_2$ (%): C, 46.38; H, 4.38; N, 33.80. Found: C, 46.36; H, 4.52; N, 33.78. MS-ESI (m/z): $[M + H]^+$ calcd for $C_8H_{10}N_5O_2$, 208.1; found, 208.1. **IR** (ATR, cm^{-1}): 1637, 1601, 1535, 1463, 1440, 1397, 1359, 1294, 1261, 1209, 1080, 1031, 1009, 971, 921, 872, 838, 796, 731, 710, 681, 647, 630, 483, 424. **^{1}H-NMR** (400 MHz, D_2O): δ (ppm) 8.54 (d, *J* = 1.4 Hz, 1H), 8.49 (s, 2H), 7.11 (s, 1H), 5.23 (dd, *J* = 9.4, 5.4 Hz, 1H), 3.63 (dd, *J* = 15.7, 5.4 Hz, 1H), 3.52 (dd, *J* = 15.7, 9.4 Hz, 1H). **^{13}C{^{1}H}-NMR** (101 MHz, D_2O): δ (ppm) 173.00 (C), 143.64 (CH), 133.79 (CH), 128.63 (C), 117.16 (CH), 60.14 (CH), 28.55 (CH_2). **$[\alpha]^{25}_D$** = +4.64° (c = 0.74, H_2O).

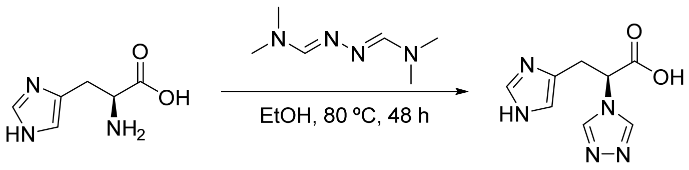

Scheme ESI2. Synthesis of (***S*-HTA**)

Synthesis of [Cu(H₂O)₂(*S*-TA)₂]·6H₂O (TAMOF-1) (known product[3])

$Cu(CH_3COO)_2{\cdot}H_2O$ (0.48 g, 2.4 mmol) was dissolved in 50 mL of H_2O, and the resulting solution was slowly added to a 100 mL aqueous solution of ***S*-HTA** (1.0 g, 4.8 mmol). The solution was stirred for 30 min, allowing for the appearance of a precipitate. The blue polycrystalline powder (**TAMOF-1**) was filtered with a Nylon membrane filter (pore size: 0.45 µm, Filter-Lab), washed with H_2O (50 × 3 mL), and dried in the air. Yield: 1.1 g (70%). Anal. Calcd for $C_{16}H_{32}CuN_{10}O_{12}$(%): C, 30.99; H, 5.2; N, 22.59. Found: C, 30.79; H, 4.83; N, 22.30. **IR** (ATR, cm^{-1}): 3289, 1615, 1532, 1495, 1395, 1355, 1263, 1242, 1211, 1180, 1116, 1087, 1015, 979, 891, 845, 752, 701, 660, 647, 549, 511, 472. Single crystals of **TAMOF-1** were obtained by slow diffusion of the $Cu(CH_3COO)_2{\cdot}H_2O$ and ***S*-HTA** solutions in an H-tube.

Screening conditions and results for the kinetic resolution of *rac*-1 and aniline (2)

Table ESI1. Screening conditions of the kinetic resolution.

Entry	Solvent	T (°C)	Conv. (%)[b]	(*R*)-**B1** (%)[c]	(*S*)-**B1** (%)[c]	(*S*)-**L1** (%)[c]	(*R*)-**L1** (%)[c]
1	-	rt	44.4	34.0	42.2	10.7	13.1
2	-	80	87.9	35.3	31.9	16.0	16.8
3	CH_2Cl_2	80	76.4	38.4	38.7	11.5	11.4
4	EtOAc	80	32.2	30.1	39.0	12.6	18.3
5	Toluene	rt	≤1.0	n.d.	n.d.	n.d.	n.d.
6	Toluene	80	10.8	24.2	44.9	11.4	19.5
7	Toluene	100	21.8	27.0	39.6	13.4	20.0
8	THF	rt	≤1.0	n.d.	n.d.	n.d.	n.d.
9	THF	60	≤1.0	n.d.	n.d.	n.d.	n.d.
10	THF	80	30.6	39.1	41.6	7.8	11.5
11[d]	ACN	0	33.0	44.4	39.3	8.4	8.0
12	ACN	rt	26.1	64.3	29.8	3.4	2.5
13	ACN	40	53.1	57.3	28.6	7.5	6.5
14	ACN	60	64.0	57.8	34.0	4.1	4.1
15	ACN	80	84.0	52.2	34.8	7.0	6.0
16	ACN	100	49.0	49.4	30.6	11.6	8.4
17[e]	ACN	40	54.0	54.1	33.8	6.4	5.7
18[f]	ACN	40	26.6	65.0	29.5	3.1	2.4
19[g]	ACN	40	24.1	54.6	24.6	12.2	8.6
20[h]	ACN	40	31.0	58.5	26.3	8.7	6.5
21[i]	ACN	60	66.7	52.2	37.8	5.2	4.8

[a] Reaction conditions: *rac*-**1** (0.5 mmol), **2** (0.5 mmol), **TAMOF-1** (10 mol%), 2 mL solvent, 16 h. No conversion was observed in absence of the catalyst. [b] Calculated by ^{1}H-NMR, using durene as a standard. [c] Calculated by SFC (Acquity UPC2, CHIRALPAK® IA column, CO_2/IPA gradient, from 0 to 40%, 1500 psi). [d] Reaction time: 3 days. [e] 1.5 eq. of **2** were used. [f] 1.5 eq. of *rac*-**1** were used. [g] 3 mol% of catalyst were used. [h] 7 mol% of catalyst were used. [i] 20 mol% of catalyst were used.

Ring-opening of *rac*-1 with *o*-aniside (3)

The catalyst (**TAMOF-1**, 10 mol%) was activated at 130 ºC *in vacuo* for at least 3 h in a Schlenk flask. The Schlenk flask was filled with nitrogen and left to cool at rt, then, a solution of *rac*-**1** (1 eq.) and **3** (1 eq.) in acetonitrile (ACN, 0.25 M) was added to the Schlenk flask and the reaction mixture was stirred under nitrogen atmosphere at 40 ºC for 16 h. The reaction mixture was left to cool at rt, then the suspension was filtered using a syringe filter with PTFE membrane (0.2 μm) and the filter was washed three times with ACN, then the crude was analyzed by ^{1}H-NMR (determination of the ratio of **4b** and **4l**) and SFC to determine the enantioselectivity.

Table ESI2. Conversion, selectivity and enantiomeric excess of **4b** and **4l**.

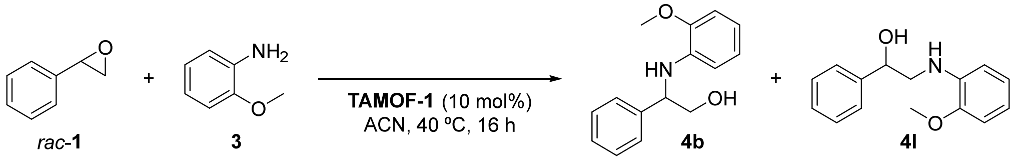

Conv. (%)[a]	Sel. 4b (%)[a]	e.e. 4b (%)[b]	Sel. 4l (%)[a]	e.e. 4l (%)[b]
50.5	74.1	56.6 (*R*)	25.9	34.8 (*S*)

[a] Calculated by ^{1}H-NMR. [b] Calculated by SFC (Acquity UPC2, CHIRALPAK® IA column, CO_2/MeOH 95:5, 1500 psi, 240 nm t_R(*S*-**4b**) = 3.56 min, t_R(*R*-**4b**) = 3.80 min, t_R(*S*-**4l**) = 6.94 min, t_R(*R*-**4l**) = 9.27 min. The absolute configuration of **4b** and **4l** was assigned by comparison of the retention times with enantiopure samples independently prepared.

Ring-opening of cyclohexene oxide (5) with aniline (2)

The catalyst (**TAMOF-1**, 10 mol%) was activated at 130 ºC *in vacuo* for at least 3 h in a Schlenk flask. The Schlenk flask was filled with nitrogen and left to cool at rt, then, a solution of **5** (1 eq.) and **2** (1 eq.) in ACN (0.25 M) was added to the Schlenk flask and the reaction mixture was stirred under nitrogen atmosphere at 40 ºC for 16 h. The reaction mixture was left to cool at rt, then the suspension was filtered using a syringe filter with PTFE membrane (0.2 μm) and the filter was washed three times with ACN, then the crude was analyzed by HPLC to determine the enantioselectivity.

Table ESI3. Conversion and enantiomeric excess of **6**.

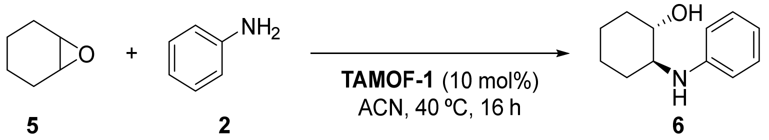

Conv. (%)[a]	e.e. 6 (%)[b]
15.0	35.4 (1*S*,2*S*)

[a] Calculated by ^{1}H-NMR. [b] Calculated by HPLC: CHIRALCEL® OD-H, *n*-Hex/IPA 85:15, 1 mL/min, 247 nm, t_R(1*S*, 2*S*-**6**) = 8.2 min (lit.[4] 10.3 min), t_R(1*R*, 2*R*-**6**) = 9.2 min (lit.[4] 11.9 min).

Synthesis of products (*R*)-B1, (*S*)-L1, BB1, LB1, LL1[5]

(*S*)-styrene oxide (3 g, 24.5 mmol) and aniline (2.73 g, 29.4 mmol) were dissolved in H_2O (40 mL, 0.625 M) and the reaction mixture was stirred for 18 h at 60 °C. The reaction mixture was extracted with EtOAc (3 x 100 mL) and the combined organic layers were combined, dried over Na_2SO_4 and concentrated *in vacuo*. The residue obtained was then purified by flash column chromatography recursively using the following solvent systems:

- DCM (100%) to eliminate aniline from the crude and to isolate **BB1** (34 mg, 0.42% yield).
- DCM/MeOH 99:1 to obtain a mixture of (*R*)-**B1** and (*S*)-**L1**, and to isolate **LB1** (97 mg, 1.19%).
- Cy/MTBE 1:1 to isolate (*R*)-**B1** (3.68 g, 70.4%), (*S*)-**L1** (193 mg, 3.69%) and **LL1** (45 mg, 0.55%).

(DCM, dichloromethane; Cy, cyclohexane; MTBE, methyl *tert*-butyl ether)

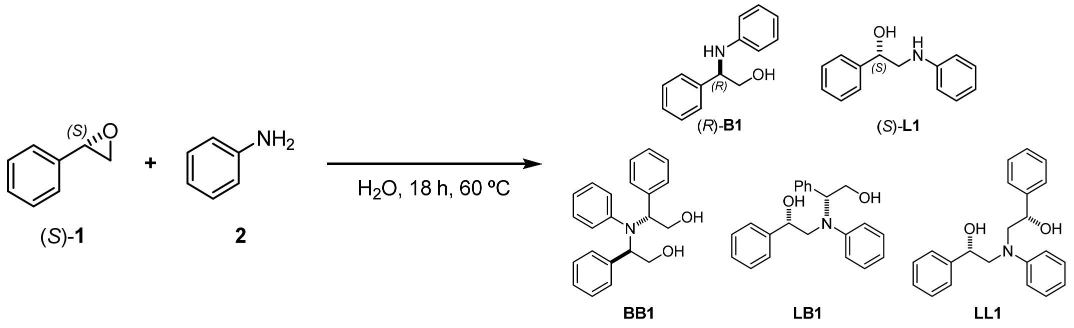

Scheme ESI3. Scheme of the synthesis of (*R*)-**B1**, (*S*)-**L1**, **BB1**, **LB1** and **LL1**

Synthesis of products LB2 and LL2

The catalyst (**TAMOF-1**, 50 mol%) was activated at 130 °C *in vacuo* for at least 3 h in a Schlenk flask. The Schlenk flask was filled with nitrogen and left to cool at rt, then, a solution of the (*R*)-styrene oxide (70 mg, 0.59 mmol) and (*S*)-**L1** (125 mg. 0.59 mmol) in ACN (2.3 mL, 0.25 M) was added to the Schlenk flask and the reaction mixture was stirred under nitrogen atmosphere at 40 °C for 70 h. The reaction mixture was left to cool at rt, then the suspension was filtered using a syringe filter with PTFE membrane (0.2 μm) and the filter was washed three times with ACN. The crude solution was concentrated *in vacuo* and the residue was purified by flash column chromatography, using first Cy/EtOAc 1:1 to isolate **BL2** as a light yellow oil (39 mg, 27.3% yield) and then Cy/MTBE 7:3 to isolate **LL2** (72.6 mg, 50.8% yield) as a light yellow oil.

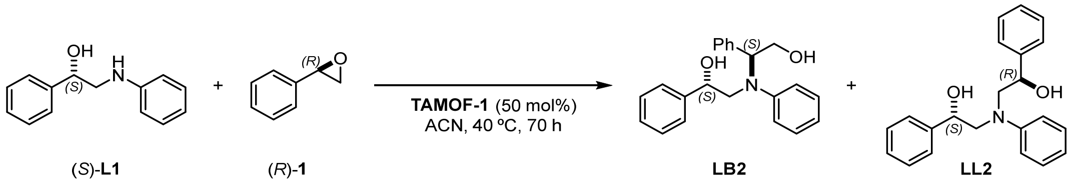

Scheme ESI4. Scheme of the synthesis of **LB2** and **LL2.**

Synthesis of *rac*-4b and *rac*-4l[6]

To 10 mL round-bottom flask, *o*-anisidine (123 mg, 1 mmol), *rac*-styrene oxide (124 mg, 1 mmol) and Schreiner's thiourea (*N,N'*-Bis[3,5(trifluoromethyl)phenyl]thiourea, 25 mg, 5 mol%) were added. The reaction mixture was stirred at 60 °C under free-solvent conditions for 2 h. The completion of the reaction was monitored by TLC (hexane/EtOAc 85:15). After completion of the reaction, the crude mixture was loaded onto a silica column and it was purified by flash column chromatography (hexane/EtOAc 85:15). *rac*-**4b** was isolated as a yellow oil and *rac*-**4l** was isolated as a pale-yellow oil; yield of *rac*-**4b** = 68% and yield of *rac*-**4l** = 12%.

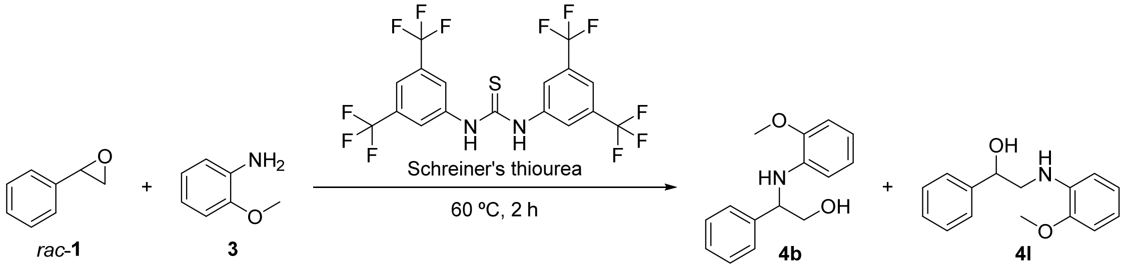

Scheme ESI5. Synthesis of the racemic mixtures of **4b** and **4l**.

Synthesis of (*S*)-4b and (*R*)-4l[7]

To an 8 mL vial equipped with a stirring bar, *o*-anisidine (185 mg, 1.5 mmol), (*R*)-styrene oxide (123 mg, 1 mmol) and zinc trifluoromethanesulfonate (37 mg, 10 mol%) were added. Then, the mixture was taken into the glovebox, where anhydrous toluene (2.5 mL, 0.4 M) was added using a micropipette. The reaction mixture was then taken outside the glovebox, heated to 100 °C and stirred for 12 h. The solvent was concentrated *in vacuo*, and the residue purified by flash column chromatography (hexane/EtOAc 85:15); yield of (*S*)-**4b** = 62% and yield of (*R*)-**4l** = 7.8%.

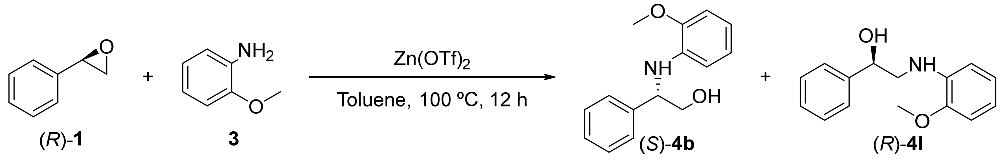

Scheme ESI6. Synthesis of (*S*)-**4b** and (*R*)-**4l**.

Synthesis of *rac-trans*-6[8]

Cyclohexene oxide (302 mg, 3.05 mmol), aniline (285 mg, 3.05 mmol) and acetic acid (0.18 mL, 3.05 mmol) were stirred together in a 1 mL vial under free-solvent conditions for 1 h. Then, K_2CO_3 (426 mg, 3.05 mmol) was added to the reaction mixture and the crude was purified by flash column chromatography (deactivated silica, hexane/EtOAc 3:1 + 3% Et_3N for deactivation, hexane/EtOAc 3:1 to elute the sample); yield = 74%.

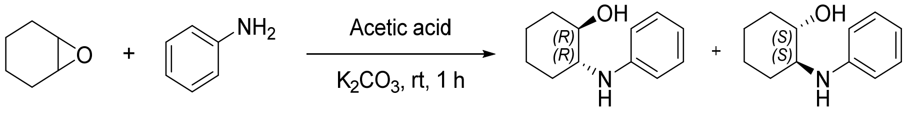

Scheme ESI7. Synthesis of *rac-trans*-**6**.

Results of the kinetic resolutions

Table ESI4. Results of the kinetic resolution with 10 mol% of **TAMOF-1.** Reaction conditions: *rac*-**1** (0.5 mmol), **2** (0.5 mmol), **TAMOF-1** (10 mol%), 2 mL ACN. Crude products of catalytic reactions were analyzed by HPLC and quantified using external standards. (CHIRALPAK® IC, *n*-Hex/IPA 99:1 to 95:5, 1 mL/min, 5 µL injection, gradient (min, %IPA): (0, 1); (7, 1); (14, 5); (40, 5). [a]Results are expressed in mol%: the sum of these amounts within a row equals to 100 mol% of the starting epoxide.

Entry	Time (h)	(*R*)-1[a]	(*S*)-1[a]	2	(*R*)-B1[a]	(*S*)-B1[a]	(*S*)-L1[a]	(*R*)-L1[a]	ee 1 (%)	ee B1 (%)	ee L1 (%)
1	0	50	50	100	0	0	0	0	0	0	0
2	4	45.6	42.5	90.2	9.0	4.4	0.9	0.6	3.5 (*R*)	34.3 (*R*)	20.0 (*S*)
3	12	34.5	27.2	66.9	20.1	11.3	1.5	1.3	11.8 (*R*)	28.0 (*R*)	7.1 (*S*)
4	17	34.0	26.6	64.7	20.6	11.9	1.5	1.4	12.2 (*R*)	26.8 (*R*)	5.3 (*S*)
5	48	24.5	14.6	46.3	31.0	19.8	1.5	1.8	25.3 (*R*)	22.0 (*R*)	-9.1 (*R*)

Entry	Time (h)	BB1[a]	LB1[a]	LL1[a]	LB2[a]	LL2[a]
1 cont.	0	0	0	0	0	0
2 cont.	4	0	0	0	0	0
3 cont.	12	0.18	0.16	0.22	0.05	0.36
4 cont.	17	0.27	0.21	0.25	0.09	0.47
5 cont.	48	0.79	0.70	0.68	0.37	1.40

Table ESI5. Results of the kinetic resolution with 50 mol% of **TAMOF-1.** Reaction conditions: *rac*-**1** (0.5 mmol), **2** (0.5 mmol), **TAMOF-1** (50 mol%), 2 mL ACN. Crude products of catalytic reactions were analyzed by HPLC and quantified using external standards. (CHIRALPAK® IC, *n*-Hex/IPA 99:1 to 95:5, 1 mL/min, 5 µL injection, gradient (min, %IPA): (0, 1); (7, 1); (14, 5); (40, 5). [a] Results are expressed in mol%: the sum of these amounts within a row equals to 100 mol% of the starting epoxide.

Entry	Time (h)	(*R*)-**1**[a]	(*S*)-**1**[a]	**2**	(*R*)-**B1**[a]	(*S*)-**B1**[a]	(*S*)-**L1**[a]	(*R*)-**L1**[a]	ee 1 (%)	ee B1 (%)	ee L1 (%)
1	0	50	50	100	0	0	0	0	0	0	0
2	1	40.6	36.3	76.5	9.7	4.7	0.9	0.6	5.6 (*R*)	34.7 (*R*)	20.0 (*S*)
3	8	30.0	21.9	59.2	23.1	14.5	1.3	1.5	15.6 (*R*)	22.9 (*R*)	-3.7 (*R*)
4	16	21.2	12.2	45.5	32.4	22.7	1.2	1.7	26.9 (*R*)	17.6 (*R*)	-17.2 (*R*)
5	24	15.6	7.1	29.2	36.0	27.0	0.9	1.6	37.4 (*R*)	14.3 (*R*)	-28.0 (*R*)
6	32	14.1	5.6	25.2	38.6	30.1	0.7	1.5	41.7 (*R*)	12.4 (*R*)	-36.4 (*R*)
7	48	5.8	1.5	23.0	42.4	36.8	0.6	1.7	58.9 (*R*)	7.1 (*R*)	-45.5 (*R*)
8	96	2.7	0.4	20.5	39.8	34.5	0.5	1.5	74.2 (*R*)	7.1 (*R*)	-57.9 (*R*)

Entry	Time (h)	BB1[a]	LB1[a]	LL1[a]	LB2[a]	LL2[a]
1 cont.	0	0	0	0	0	0
2 cont.	1	0	0	0	0	0
3 cont.	8	0.02	0	0.41	0.08	0.47
4 cont.	16	0.20	0.08	0.59	0.14	0.99
5 cont.	24	0.64	0.08	0.86	0.26	1.57
6 cont.	32	0.79	0.16	1.00	0.29	1.84
7 cont.	48	1.10	0.22	1.04	0.73	2.38
8 cont.	96	1.73	0.46	1.09	1.08	2.62

Characterization of amino alcohols and aminodiols

(*R*)-B1 (known product[9])

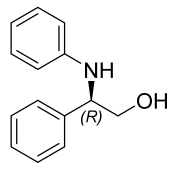

(*R*)-B1

^{1}H NMR (400 MHz, $CDCl_3$) δ 7.41 – 7.31 (m, 4H), 7.31 – 7.24 (m, 1H), 7.16 – 7.05 (m, 2H), 6.75 – 6.65 (m, 1H), 6.63 – 6.55 (m, 2H), 4.51 (dd, *J* = 7.0, 4.2 Hz, 1H), 3.95 (dd, *J* = 11.1, 4.2 Hz, 1H), 3.76 (dd, *J* = 11.1, 7.0 Hz, 1H). **^{13}C{^{1}H} NMR** (101 MHz, $CDCl_3$) δ 147.37 (C), 140.25 (C), 129.30 (CH), 128.99 (CH), 127.77 (CH), 126.87 (CH), 118.05 (CH), 114.00 (CH), 67.53 (CH), 60.02 (CH_2). **HRMS-ESI** (*m/z*): [M + H]$^+$ calcd for $C_{14}H_{16}NO$, 214.1226; found, 214.1219. **$[\alpha]^{25}_D$** = –21.9 (c = 0.5, $CHCl_3$), lit. (*S*)-B1 = +27.5 (c = 0.5, $CHCl_3$); **IR** (neat, cm^{-1}) $\bar{\upsilon}$ 3396, 3053, 3025, 2927, 2873, 1600, 1502, 1451, 1430, 1353, 1315, 1264, 1180, 1064, 1027, 749, 693. HPLC (CHIRALPAK® IC, *n*-Hex/IPA 99:1 to 95:5, 1 mL/min, 5 µL injection, gradient (min, %IPA): (0, 1); (7, 1); (14, 5); (40, 5)): t_R(*R*)-**B1**= 20.4 min, t_R(*S*)-**B1**= 28.4 min.

(*S*)-L1 (known product[10])

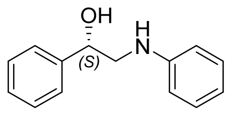

(*S*)-L1

^{1}H NMR (400 MHz, $CDCl_3$) δ 7.48 – 7.39 (m, 4H), 7.39 – 7.31 (m, 1H), 7.28 – 7.18 (m, 2H), 6.78 (tt, *J* = 7.3, 1.1 Hz, 1H), 6.73 – 6.67 (m, 2H), 4.96 (dd, *J* = 8.4, 3.2 Hz, 1H), 4.07 (s, 1H), 3.47 (dd, *J* = 13.1, 3.9 Hz, 1H), 3.33 (dd, *J* = 13.1, 8.4 Hz, 1H), 2.40 (d, *J* = 3.3 Hz, 1H). **^{13}C{^{1}H} NMR** (101 MHz, $CDCl_3$) δ 148.02 (C), 142.14 (C), 129.49 (CH), 128.80 (CH), 128.17 (CH), 126.03 (CH), 118.28 (CH), 113.60 (CH), 72.65 (CH), 51.95 (CH_2). **HRMS-ESI** (*m/z*): [M + H]$^+$ calcd for $C_{14}H_{16}NO$, 214.1226; found, 214.1220. **$[\alpha]^{25}_D$** = –19.5 (c = 0.97, acetone), lit. (*R*)-L1 = +17.2 (c = 0.98, acetone). **IR** (neat, cm^{-1}) $\bar{\upsilon}$ 3393, 3053, 3026, 2923, 2823, 1602, 1504, 1453, 1432, 1317, 1259, 1058, 750, 693. HPLC (CHIRALPAK® IC, *n*-Hex/IPA 99:1 to 95:5, 1 mL/min, 5 µL injection, gradient (min, %IPA): (0, 1); (7, 1); (14, 5); (40, 5)): t_R(*S*)-**L1**= 22.9 min, t_R(*R*)-**L1**= 21.9 min.

BB1

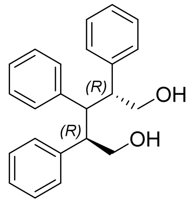

BB1

^{1}H NMR (500 MHz, $CDCl_3$) δ 7.30 – 7.25 (m, 6H), 7.22 (dd, *J* = 8.4, 7.2 Hz, 2H), 7.15 – 7.07 (m, 5H), 6.93 (d, *J* = 7.9 Hz, 2H), 4.57 (dd, *J* = 7.9, 5.7 Hz, 2H)23.89 (dd, *J* = 11.2, 7.9 Hz, 2H), 3.69 (dd, *J* = 11.3, 5.7 Hz, 2H), 2.31 (s, 2H). **^{13}C{^{1}H} NMR** (126 MHz, $CDCl_3$) δ 144.61 (C), 138.21 (C), 128.89 (CH), 128.57 (CH), 128.53 (CH), 128.02 (CH), 126.64 (CH), 124.41 (CH), 64.23 (CH), 63.29 (CH_2). **HRMS-ESI** (*m/z*): [M + H]$^+$ calcd for $C_{22}H_{24}NO_2$, 334.1802; found, 334.1787. **$[\alpha]^{25}_D$** = –30.53 (c = 0.16, CH_2Cl_2). **IR** (neat, cm^{-1}) $\bar{\upsilon}$ 3345, 3060, 2926, 1596, 1499, 1453, 1404, 1263, 1149, 1025, 735, 697. HPLC (CHIRALPAK® IC, *n*-Hex/IPA 99:1 to 95:5, 1 mL/min, 5 µL injection, gradient (min, %IPA): (0, 1); (7, 1); (14, 5); (40, 5)): t_R = 36.2 min.

LB1

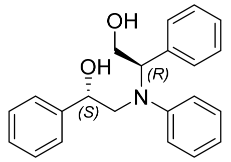

LB1

^{1}**H NMR** (400 MHz, $CDCl_3$) δ 7.40 – 7.27 (m, 10H), 7.10 – 7.04 (m, 2H), 6.98 (d, *J* = 8.1 Hz, 2H), 6.92 (t, *J* = 7.3 Hz, 1H), 4.94 (t, *J* = 6.7 Hz, 1H), 4.86 (t, *J* = 6.7 Hz, 1H), 4.00 – 3.89 (m, 2H), 3.53 (s, 1H), 3.39 (d, *J* = 6.7 Hz, 2H), 2.49 (s, 1H). ^{13}**C{^{1}H} NMR** (101 MHz, $CDCl_3$) δ 148.80 (C), 142.70 (C), 137.61 (C), 129.21 (CH), 128.68 (CH), 128.66 (CH), 128.16 (CH), 127.59 (CH), 127.16 (CH), 126.00 (CH), 119.97 (CH), 118.44 (CH), 71.73 (CH), 68.13 (CH), 61.64 (CH_2), 56.73 (CH_2). **HRMS-ESI** (*m/z*): $[M + H]^+$ calcd for $C_{22}H_{24}NO_2$, 334.1802; found, 334.1792. **$[\alpha]^{25}_{D}$** = –26.29 (c = 0.25, CH_2Cl_2). **IR** (neat, cm^{-1}) $\bar{\upsilon}$ 3360, 3060, 3028, 2923, 1597, 1495, 1451, 1349, 1264, 1195, 1028, 915, 804, 750, 698. HPLC (CHIRALPAK® IC, *n*-Hex/IPA 99:1 to 95:5, 1 mL/min, 5 µL injection, gradient (min, %IPA): (0, 1); (7, 1); (14, 5); (40, 5)): t_R = 29.7 min.

LL1

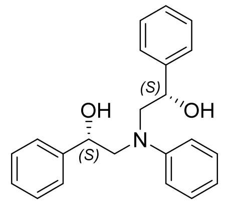

LL1

^{1}**H NMR** (400 MHz, $CDCl_3$) δ 7.47 – 7.36 (m, 8H), 7.35 – 7.27 (m, 4H), 6.96 (d, *J* = 8.1 Hz, 2H), 6.82 (t, *J* = 7.3 Hz, 1H), 5.06 (dd, *J* = 9.6, 3.4 Hz, 2H), 3.61 (dd, *J* = 15.0, 3.4 Hz, 2H), 3.51 (dd, *J* = 15.0, 9.6 Hz, 2H), 3.19 (s, 2H). ^{13}**C{^{1}H} NMR** (101 MHz, $CDCl_3$) δ 148.61 (C), 142.03 (C), 129.44 (CH), 128.77 (CH), 128.04 (CH), 126.03 (CH), 118.22 (CH), 114.64 (CH), 71.39 (CH), 60.60 (CH_2). **HRMS-ESI** (*m/z*): $[M + H]^+$ calcd for $C_{22}H_{24}NO_2$, 334.1802; found, 334.1793. **$[\alpha]^{25}_{D}$** = –38.98 (c = 0.31, CH_2Cl_2). **IR** (neat, cm^{-1}) $\bar{\upsilon}$ 3362, 3061, 3028, 2920, 2853, 1597, 1503, 1452, 1346, 1262, 1195, 1171, 1090, 1059, 1027, 994, 745, 698. HPLC (CHIRALPAK® IC, *n*-Hex/IPA 99:1 to 95:5, 1 mL/min, 5 µL injection, gradient (min, %IPA): (0, 1); (7, 1); (14, 5); (40, 5)): t_R = 25.1 min.

LB2

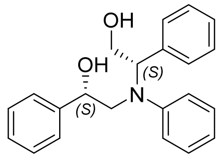

LB2

^{1}**H NMR** (400 MHz, $CDCl_3$) δ 7.40 – 7.27 (m, 7H), 7.25 – 7.15 (m, 3H), 7.13 – 7.04 (m, 4H), 6.86 (t, *J* = 7.3 Hz, 1H), 5.21 (dd, *J* = 10.4, 4.8 Hz, 1H), 5.03 (dd, *J* = 10.4, 2.1 Hz, 1H), 4.29 – 4.13 (m, 2H), 3.40 (dd, *J* = 15.3, 2.1 Hz, 1H), 3.14 (dd, *J* = 15.3, 10.4 Hz, 1H). ^{13}**C{^{1}H} NMR** (101 MHz, $CDCl_3$) δ 149.29 (C), 142.17 (C), 138.83 (C), 129.56 (CH), 128.82 (CH), 128.65 (CH), 128.20 (CH), 127.43 (CH), 127.04 (CH), 125.84 (CH), 118.88 (CH), 116.32 (CH), 70.57 (CH), 65.04 (CH), 62.00 (CH_2), 52.67 (CH_2). **HRMS-ESI** (*m/z*): $[M + H]^+$ calcd for $C_{22}H_{24}NO_2$, 334.1802; found, 334.1816. **$[\alpha]^{25}_{D}$** = –51.01 (c = 0.34, CH_2Cl_2). **IR** (neat, cm^{-1}) $\bar{\upsilon}$ 3299, 3061, 3030, 2961, 2915, 1633, 1597, 1498, 1450, 1383, 1344, 1190, 1157, 1060, 1031, 751, 697. HPLC (CHIRALPAK® IC, *n*-Hex/IPA 99:1 to 95:5, 1 mL/min, 5 µL injection, gradient (min, %IPA): (0, 1); (7, 1); (14, 5); (40, 5)): t_R = 27.3 min.

LL2

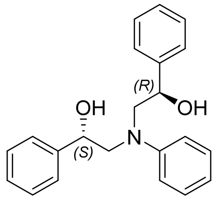

LL2

^{1}H NMR (400 MHz, $CDCl_3$) δ 7.49 – 7.28 (m, 12H), 6.86 (d, *J* = 7.4 Hz, 3H), 5.10 (dd, *J* = 9.8, 2.2 Hz, 2H), 4.51 (s, 2H), 3.82 (dd, *J* = 15.3, 2.2 Hz, 2H), 3.38 (dd, *J* = 15.3, 9.8 Hz, 2H). **^{13}C{^{1}H} NMR** (101 MHz, $CDCl_3$) δ 147.90 (C), 141.93 (C), 129.67 (CH), 128.79 (CH), 128.10 (CH), 125.98 (CH), 117.59 (CH), 112.93 (CH), 72.78 (CH), 63.23 (CH_2). **HRMS-ESI** (*m/z*): $[M + H]^+$ calcd for $C_{22}H_{24}NO_2$, 334.1802; found, 334.1813. **IR** (neat, cm^{-1}) $\bar{\upsilon}$ 3326, 3060, 3028, 2893, 1597, 1502, 1452, 1344, 1196, 1168, 1057, 993, 745, 678. HPLC (CHIRALPAK® IC, *n*-Hex/IPA 99:1 to 95:5, 1 mL/min, 5 µL injection, gradient (min, %IPA): (0, 1); (7, 1); (14, 5); (40, 5)): t_R = 24.4 min]

(*S*)-4b (known product[7])

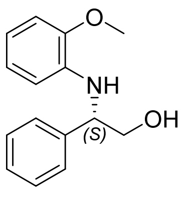

(*S*)-4b

^{1}H NMR (400 MHz, $CDCl_3$) δ 7.39 – 7.30 (m, 4H), 7.28 – 7.23 (m, 1H), 6.78 (dd, *J* = 7.7, 1.6 Hz, 1H), 6.71 (td, *J* = 7.6, 1.6 Hz, 1H), 6.65 (td, *J* = 7.6, 1.7 Hz, 1H), 6.41 (dd, *J* = 7.7, 1.7 Hz, 1H), 5.00 (s, 1H), 4.54 (dd, *J* = 7.3, 4.3 Hz), 3.96 (ddd, *J* = 11.5, 7.3, 4.5 Hz, 1H), 3.90 (s, 3H), 3.81 (ddd, *J* = 11.5, 7.3, 4.5 Hz, 1H), 1.80 (dd, *J* = 7.3, 5.1 Hz, 1H). **^{13}C{[1]H. NMR** (101 MHz, $CDCl_3$) δ 147.29 (C), 140.35 (C), 137.14 (C), 128.93 (CH), 127.70 (CH), 126.85 (CH), 121.25 (CH), 117.27 (CH), 111.61 (CH), 109.58 (CH), 67.64 (CH), 59.93 (CH_2), 55.62 (CH_3). **MS-ESI** (*m/z*): $[M + H]^+$ = 244.1. **$[\alpha]^{25}_D$** = –2.5 (c = 0.60, $CHCl_3$), lit. –30.0 (c = 0.60, $CHCl_3$). SFC (Acquity UPC2, CHIRALPAK® IA column, CO_2/MeOH 95:5, 1500 psi, 240 nm): t_R(*S*)-**4b** = 3.6 min, t_R(*R*)-**4b** = 6.8 min.

(*R*)-4l (known product[7])

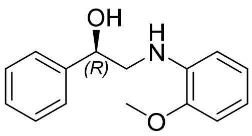

(*R*)-4l

^{1}H NMR (400 MHz, $CDCl_3$) δ 7.46 – 7.36 (m, 4H), 7.35 – 7.30 (m, 1H), 6.88 (td, *J* = 7.6, 1.5 Hz, 1H), 6.80 (dd, *J* = 8.0, 1.3 Hz, 1H), 6.76 – 6.68 (m, 2H), 4.96 (dd, *J* = 8.9, 3.7 Hz, 1H), 4.66 (s, 1H), 3.86 (s, 3H), 3.45 (dd, *J* = 13.2, 3.7 Hz, 1H), 3.32 (dd, *J* = 13.2, 8.9 Hz, 1H), 2.50 (s, 1H). **^{13}C{^{1}H} NMR** (101 MHz, $CDCl_3$) δ 147.36 (C), 142.21 (C), 137.94 (C), 128.72 (CH), 128.05 (CH), 126.04 (CH), 121.41 (CH), 117.43 (CH), 110.63 (CH), 109.85 (CH), 72.68 (CH), 55.62 (CH_2), 52.01 (CH_3). **MS-ESI** (*m/z*): $[M + H]^+$ = 244.2. **$[\alpha]^{25}_D$** = –2.9 (c = 0.35, $CHCl_3$), lit. –2.6 (c = 0.35, $CHCl_3$). SFC (Acquity UPC2, CHIRALPAK® IA column, CO_2/MeOH 95:5, 1500 psi, 240 nm): t_R(*R*)-**4l** = 9.4 min, t_R(*S*)-**4l** = 6.9 min.

rac-trans-6 (known product[8])

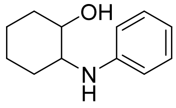

rac-trans-**6**

^{1}H NMR (300 MHz, $CDCl_3$) δ 7.23 – 7.14 (m, 2H), 6.79 – 6.68 (m, 2H), 3.43 – 3.22 (m, 2H), 3.22 – 3.07 (m, 1H), 2.72 (s, 1H), 2.18 – 2.07 (m, 2H), 1.83 – 1.65 (m, 2H), 1.49 – 1.20 (m, 3H), 1.16 – 0.96 (m, 1H). **^{13}C{^{1}H} NMR** (75 MHz, $CDCl_3$) δ 147.96 (C), 129.49 (CH),

118.54 (CH), 114.54 (CH), 74.73 (CH), 60.34 (CH), 33.28 (CH_2), 31.79 (CH_2), 25.20 (CH_2), 24.42 (CH_2). **MS-ESI** (*m/z*): $[M + H]^+$ = 192.2. HPLC (CHIRALCEL® OD-H, *n*-Hex/IPA 85:15, 1 mL/min, 247 nm): t_R(1*S*, 2*S*-**6**) = 8.2 min (lit.[4] 10.3 min) and t_R(1*R*, 2*R*-**6**)= 9.2 min (lit.[4] 11.9 min).

NMR spectra

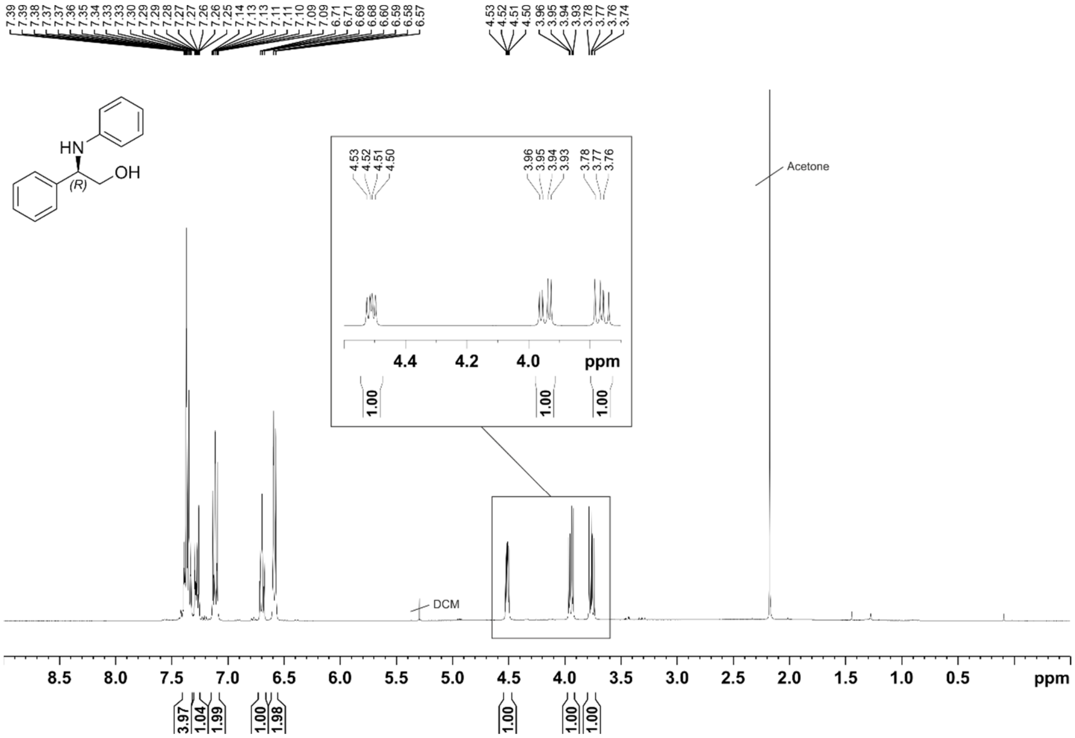

Figure ESI1. ^{1}H-NMR (400 MHz, $CDCl_3$) spectra of isolated compound (*R*)-**B1**

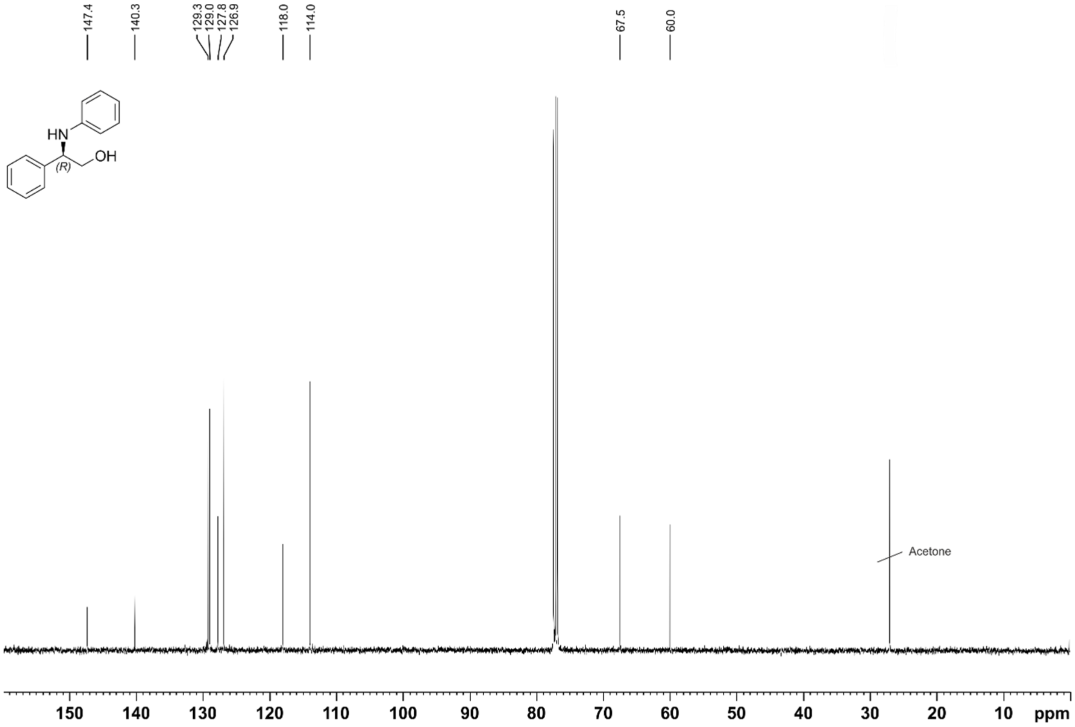

Figure ESI2. $^{13}C\{^{1}H\}$-NMR (101 MHz, $CDCl_3$) spectra of isolated compound (*R*)-**B1**

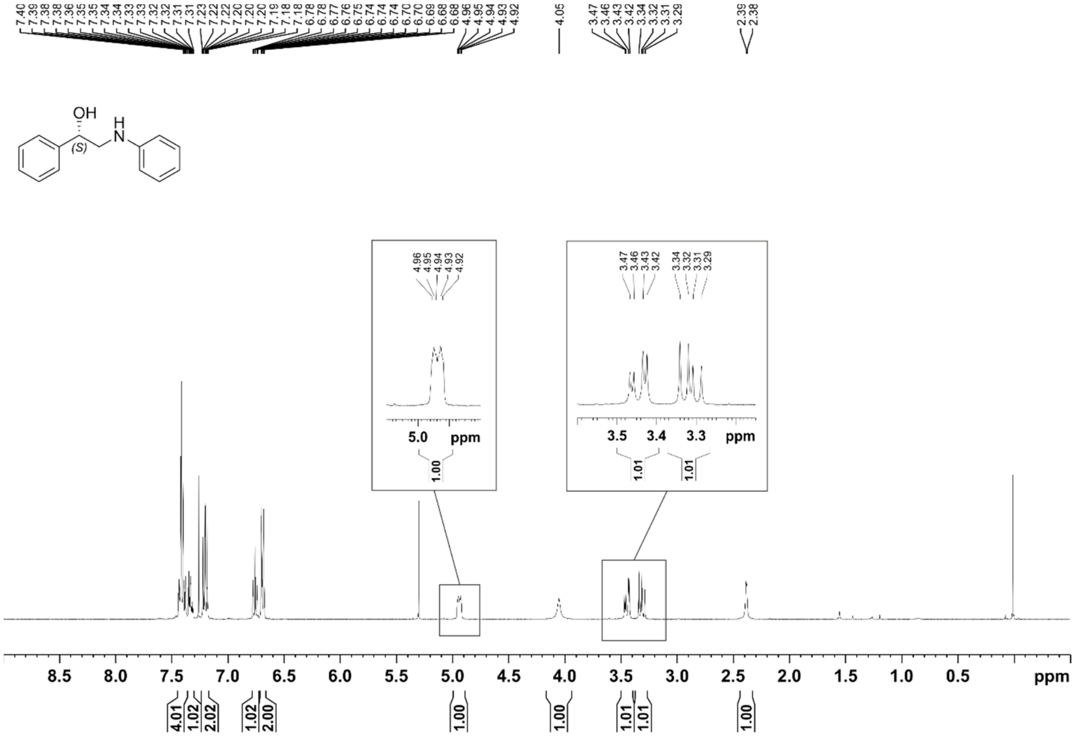

Figure ESI3. ^{1}H-NMR (400 MHz, $CDCl_3$) spectra of isolated compound (*S*)-**L1**

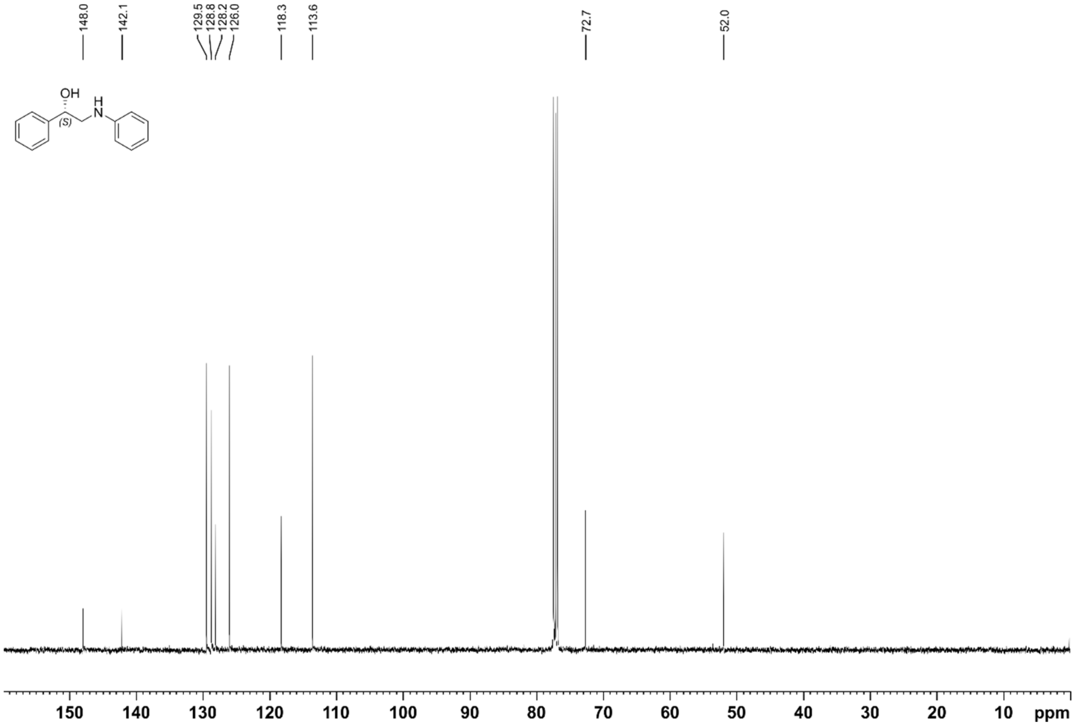

Figure ESI4. $^{13}C\{^{1}H\}$-NMR (101 MHz, $CDCl_3$) spectra of isolated compound (*S*)-**L1**

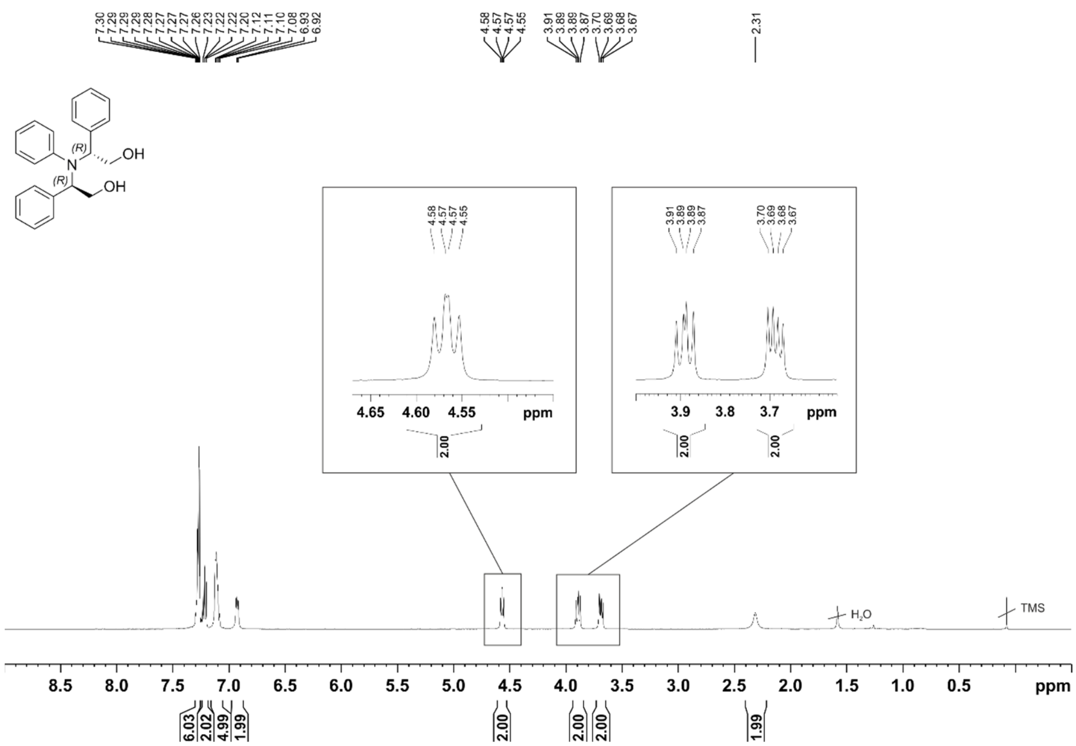

Figure ESI5. ^{1}H-NMR (500 MHz, $CDCl_3$) spectra of isolated compound **BB1**

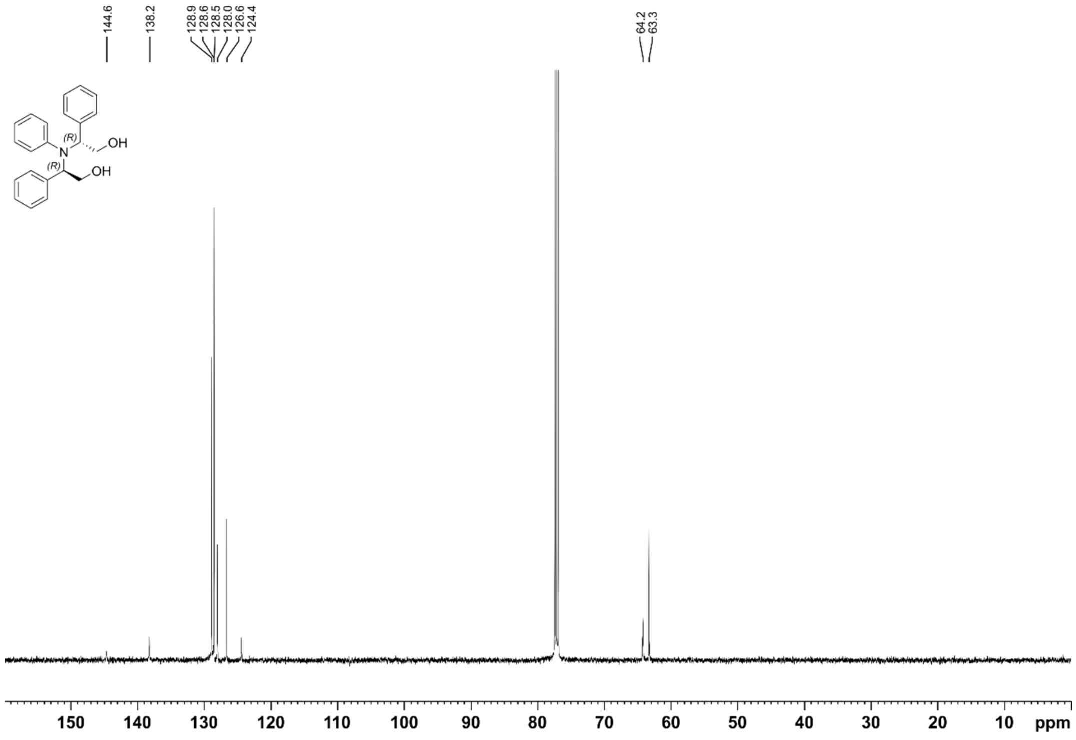

Figure ESI6. $^{13}C\{^{1}H\}$-NMR (126 MHz, $CDCl_3$) spectra of isolated compound **BB1**

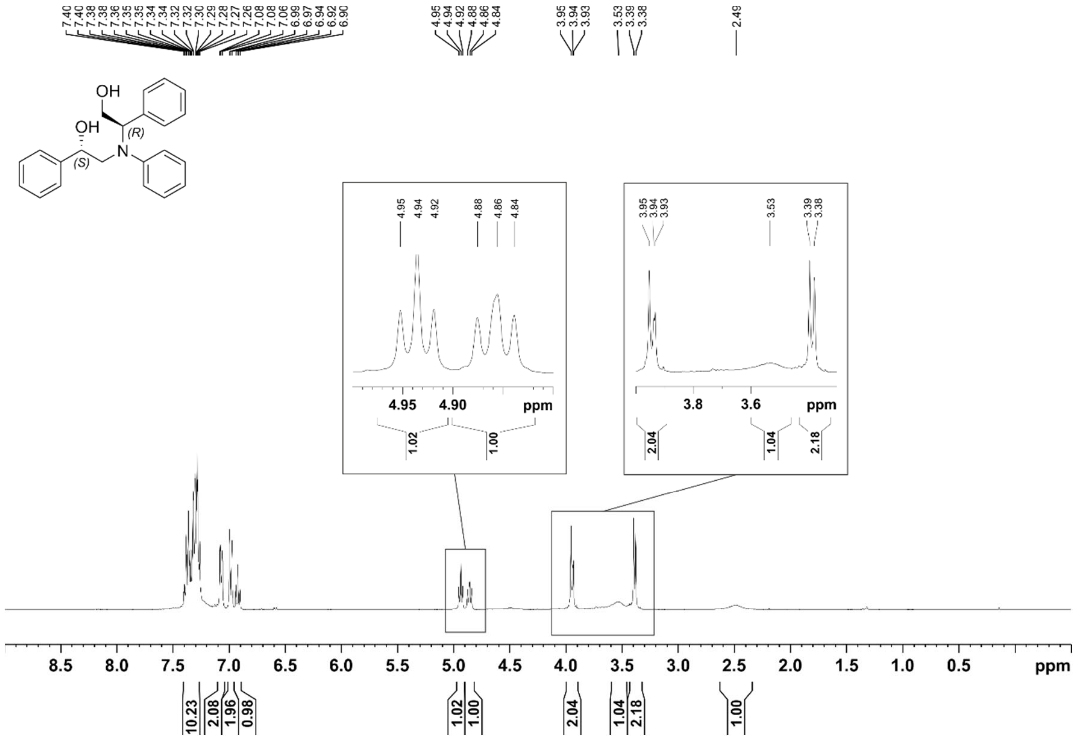

Figure ESI7. ^{1}H-NMR (400 MHz, $CDCl_3$) spectra of isolated compound **LB1**

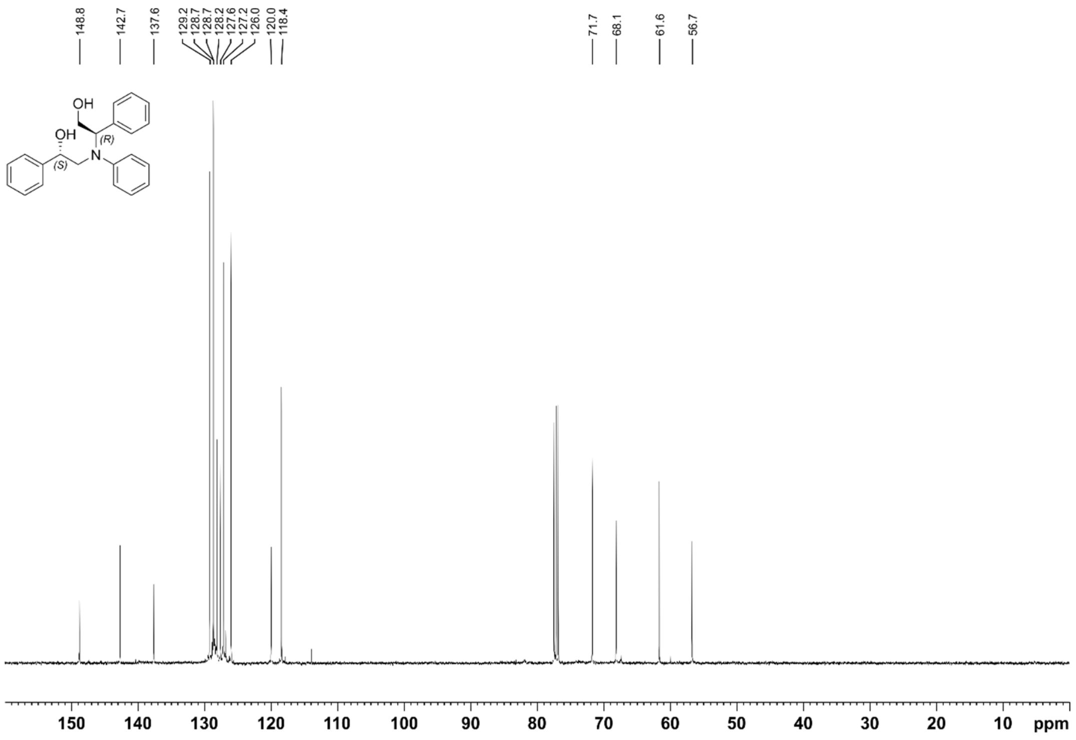

Figure ESI8. $^{13}C\{^{1}H\}$-NMR (101 MHz, $CDCl_3$) spectra of isolated compound **LB1**

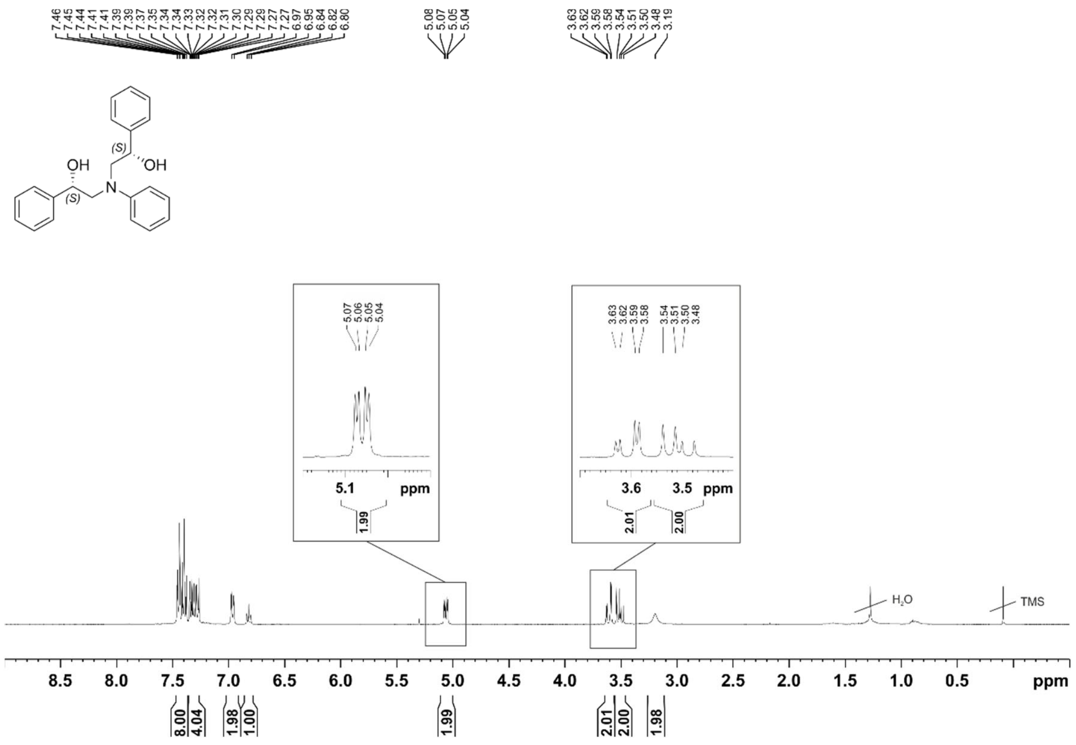

Figure ESI9. ^{1}H-NMR (400 MHz, $CDCl_3$) spectra of isolated compound **LL1**

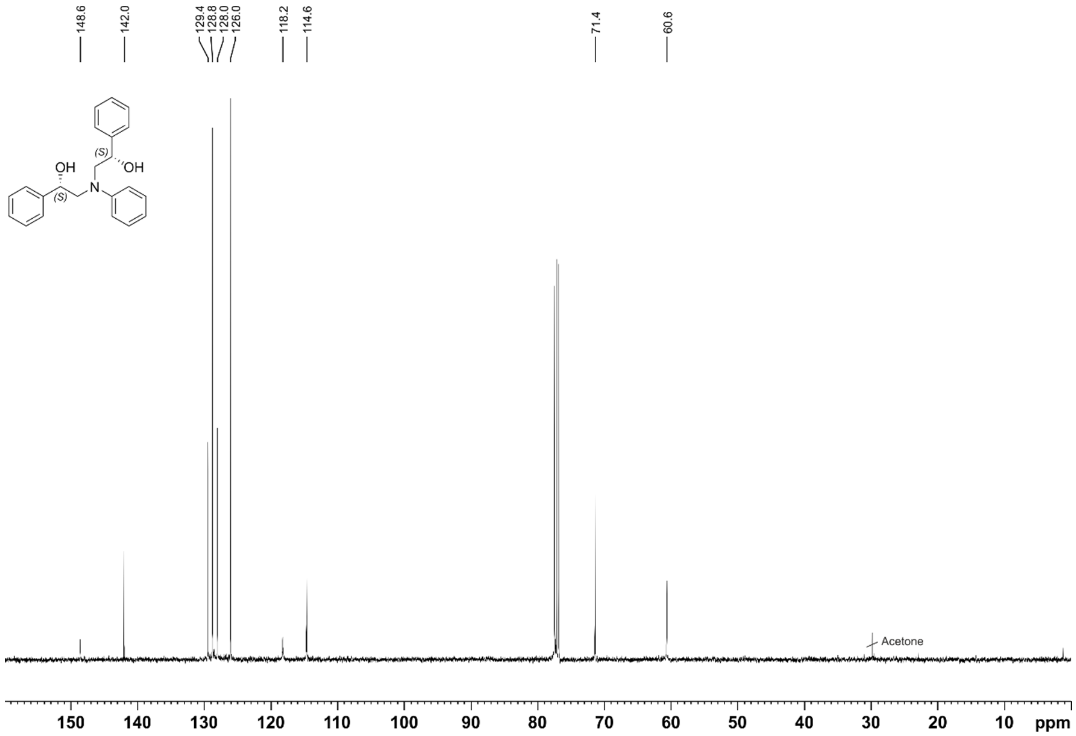

Figure ESI10. $^{13}C\{^{1}H\}$-NMR (101 MHz, $CDCl_3$) spectra of isolated compound **LL1**

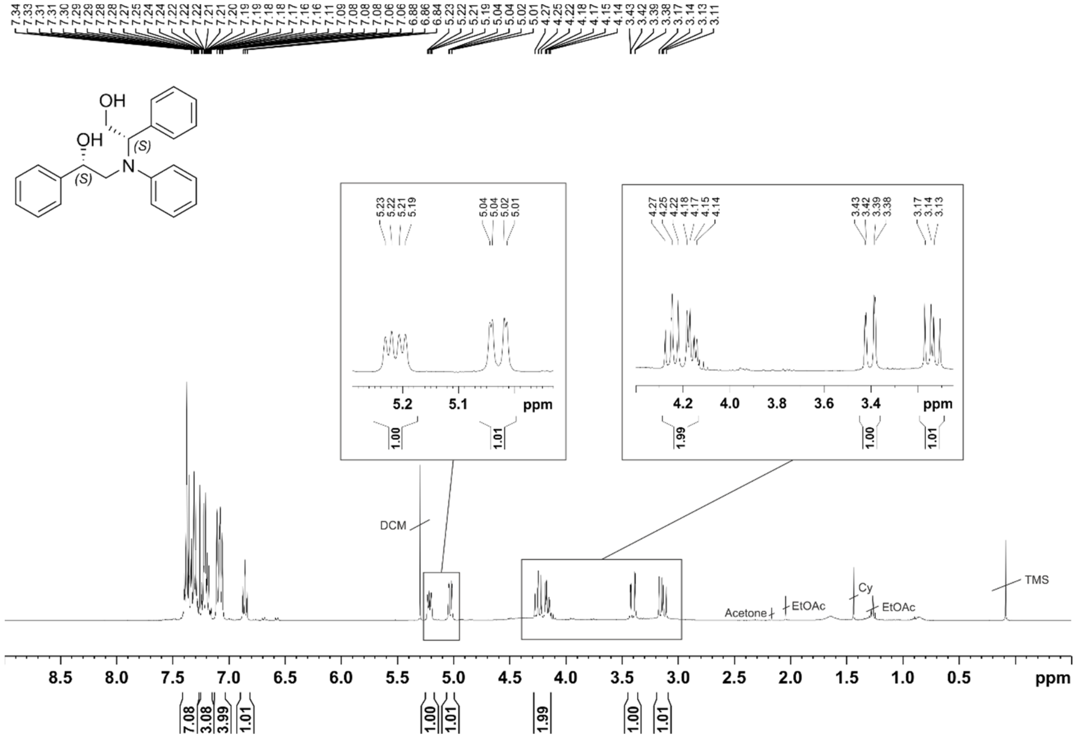

Figure ESI11. ^{1}H-NMR (400 MHz, $CDCl_3$) spectra of isolated compound **LB2**

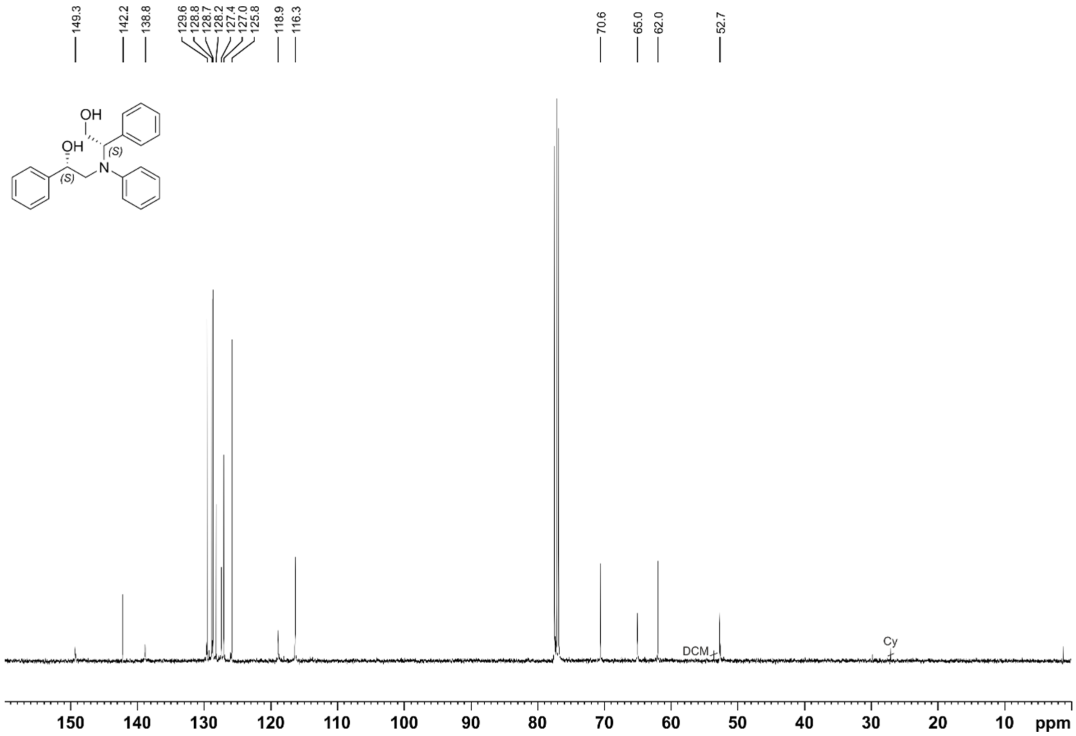

Figure ESI12. ^{13}C{^{1}H}-NMR (101 MHz, $CDCl_3$) spectra of isolated compound **LB2**

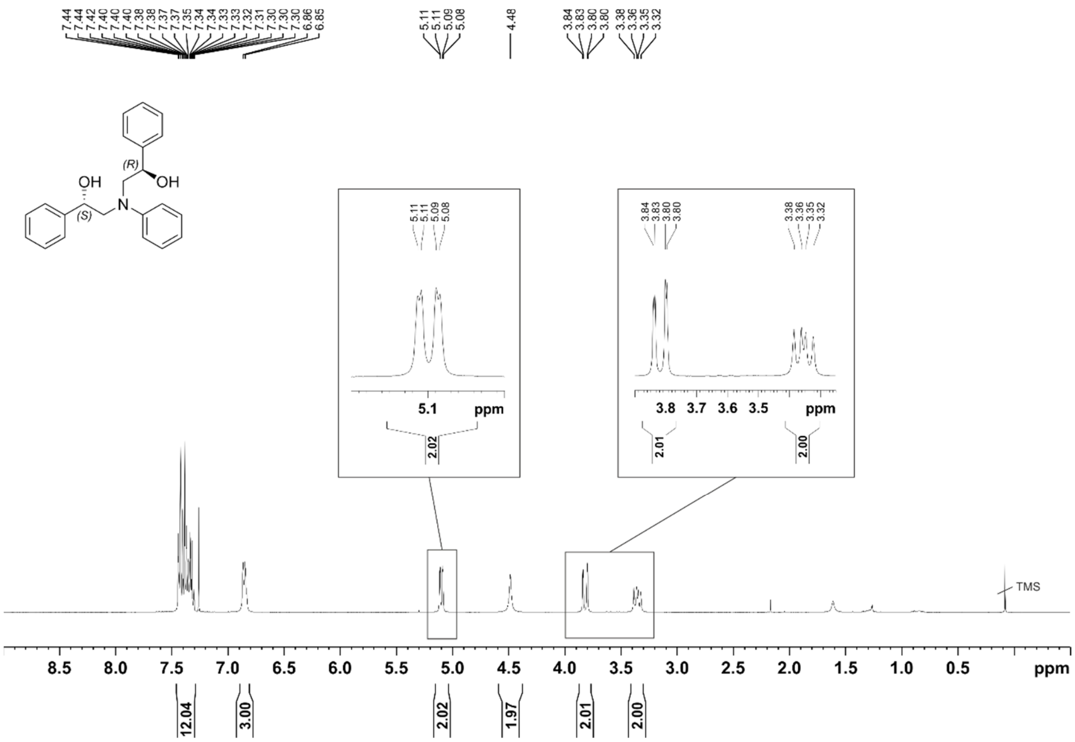

Figure ESI13. ^{1}H-NMR (400 MHz, $CDCl_3$) spectra of isolated compound **LL2**

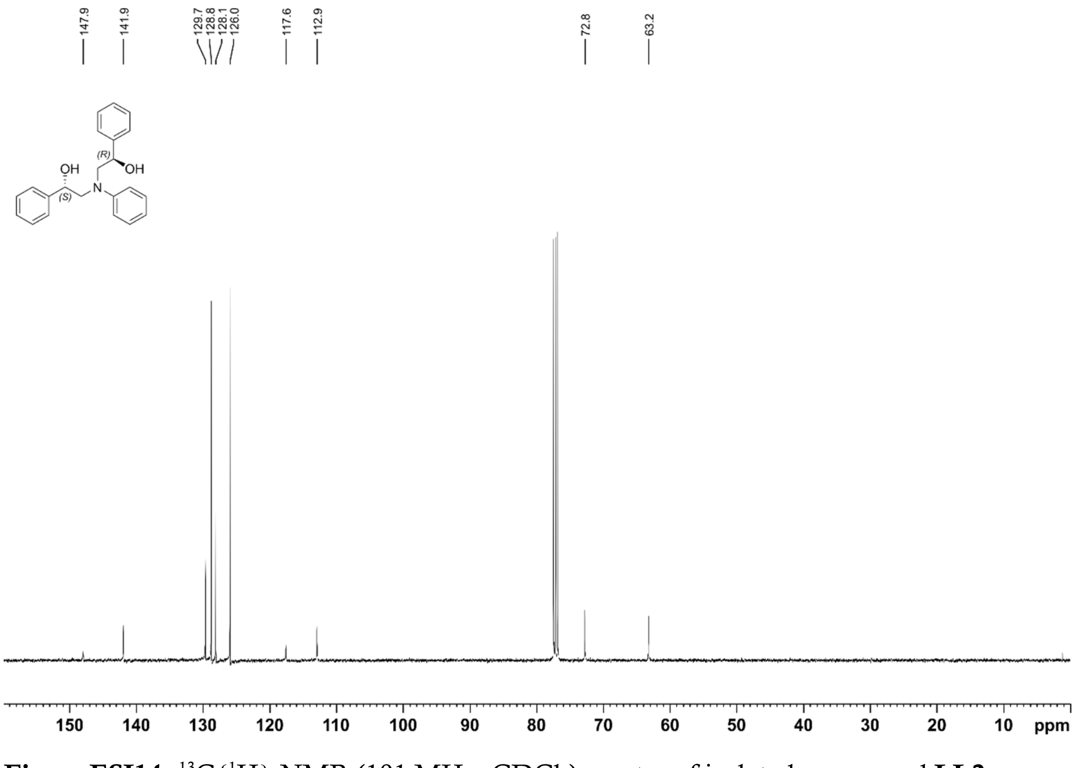

Figure ESI14. ^{13}C{^{1}H}-NMR (101 MHz, $CDCl_3$) spectra of isolated compound **LL2**

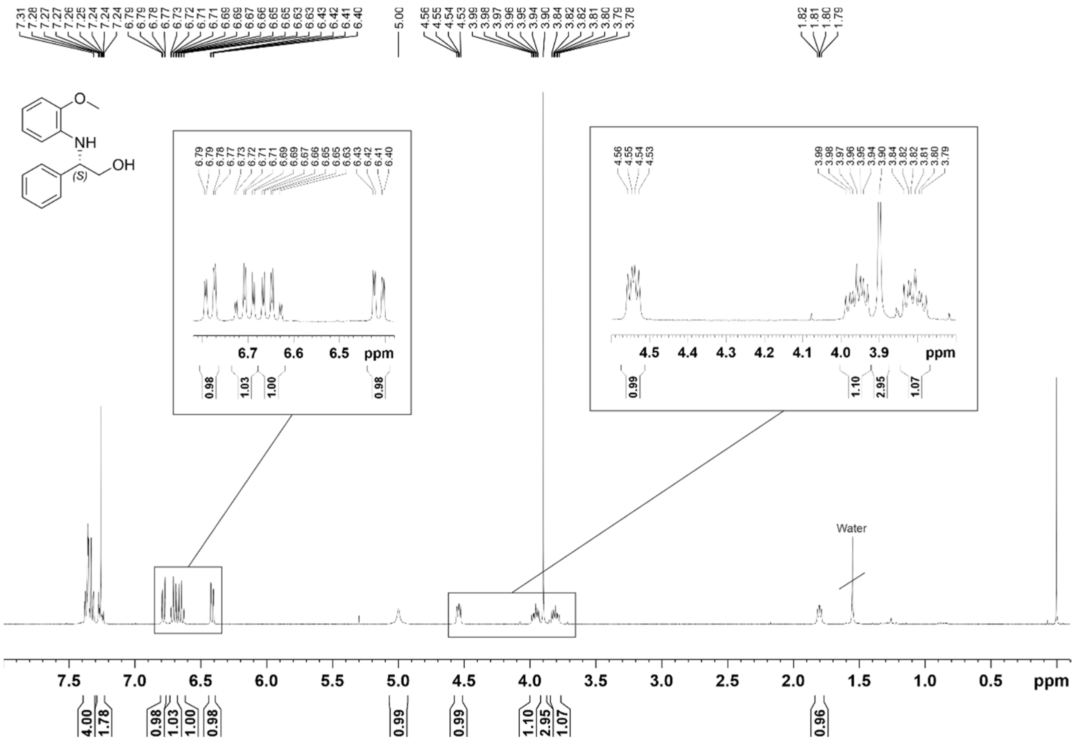

Figure ESI15. ^{1}H-NMR (400 MHz, $CDCl_3$) spectra of isolated compound (*S*)-**4b**

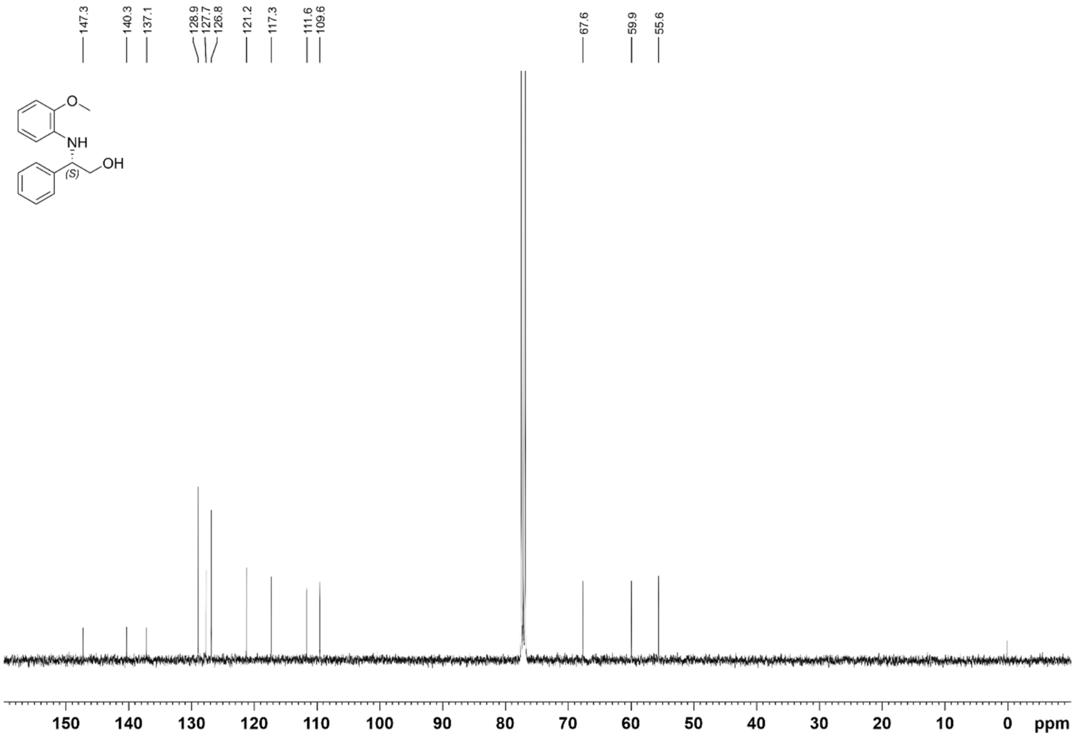

Figure ESI16. $^{13}C\{^{1}H\}$-NMR (101 MHz, $CDCl_3$) spectra of isolated compound (*S*)-**4b**

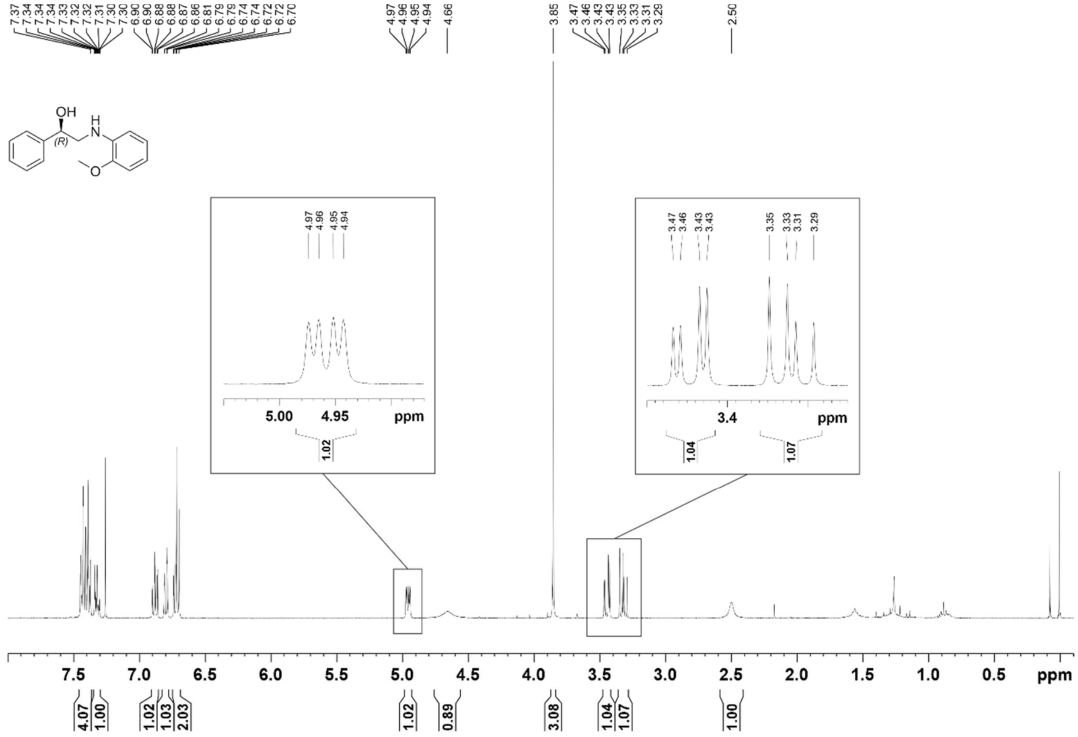

Figure ESI17. ^{1}H-NMR (400 MHz, $CDCl_3$) spectra of isolated compound (*R*)-**4l**

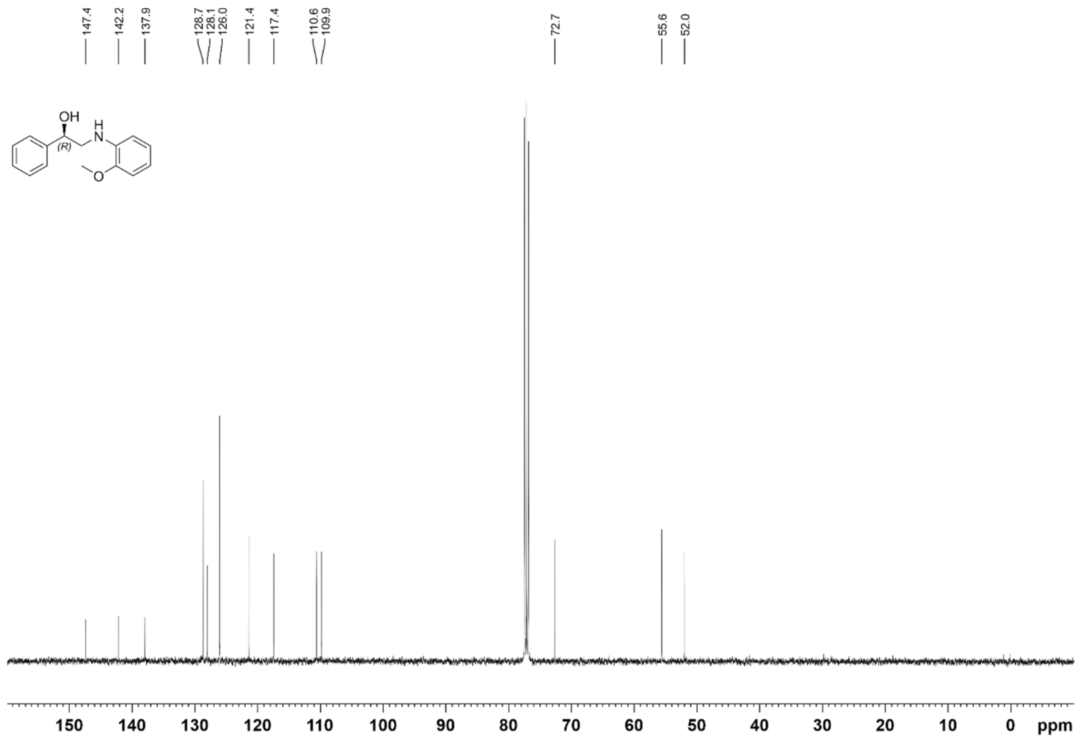

Figure ESI18. $^{13}C\{^{1}H\}$-NMR (101 MHz, $CDCl_3$) spectra of isolated compound (*R*)-**4l**

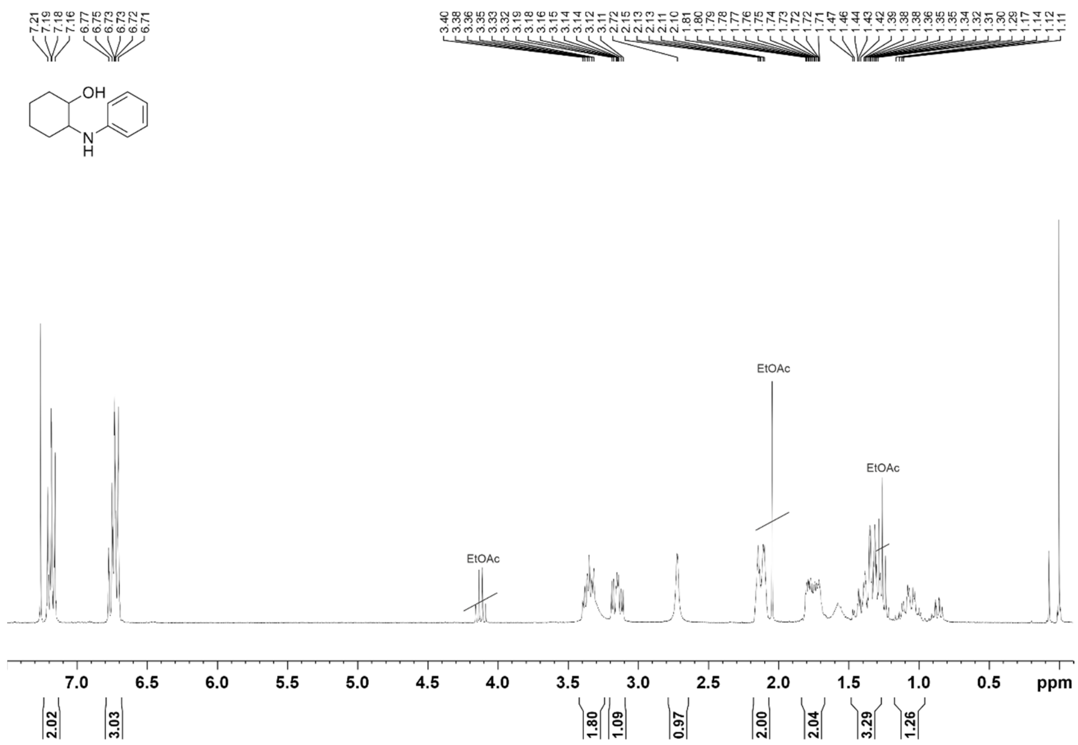

Figure ESI19. ^{1}H-NMR (300 MHz, $CDCl_3$) spectra of compound *rac-trans*-**6**

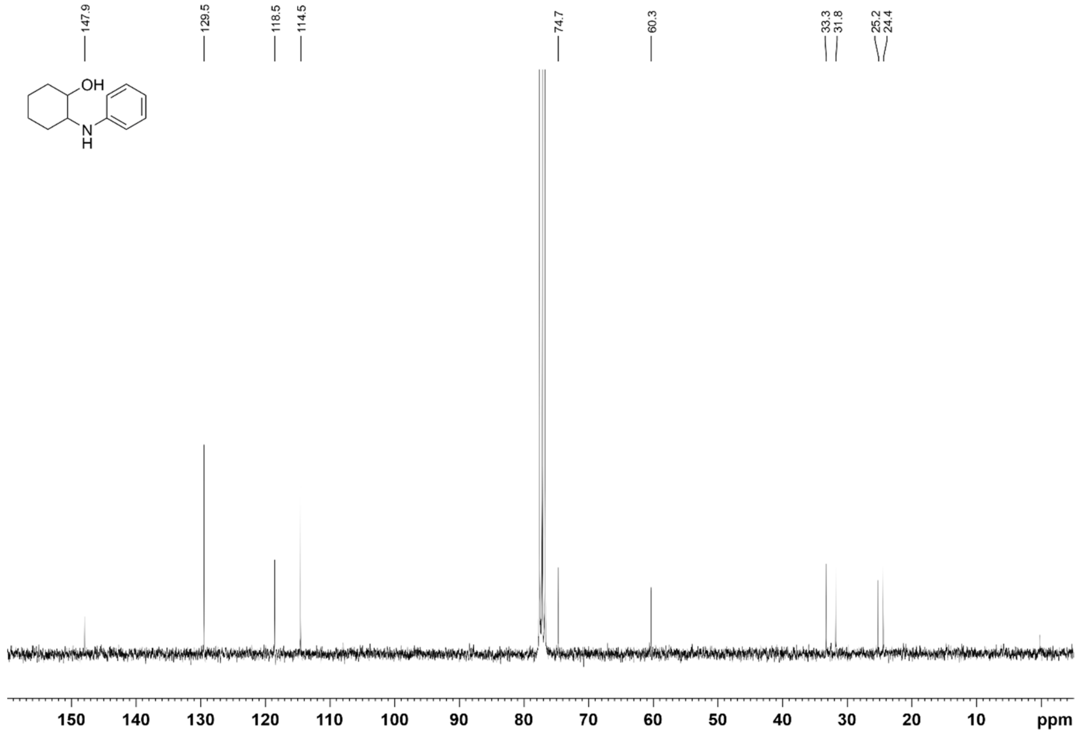

Figure ESI20. ^{13}C{^{1}H}-NMR (75 MHz, $CDCl_3$) spectra of compound *rac-trans*-**6**

HPLC and SFC chromatograms

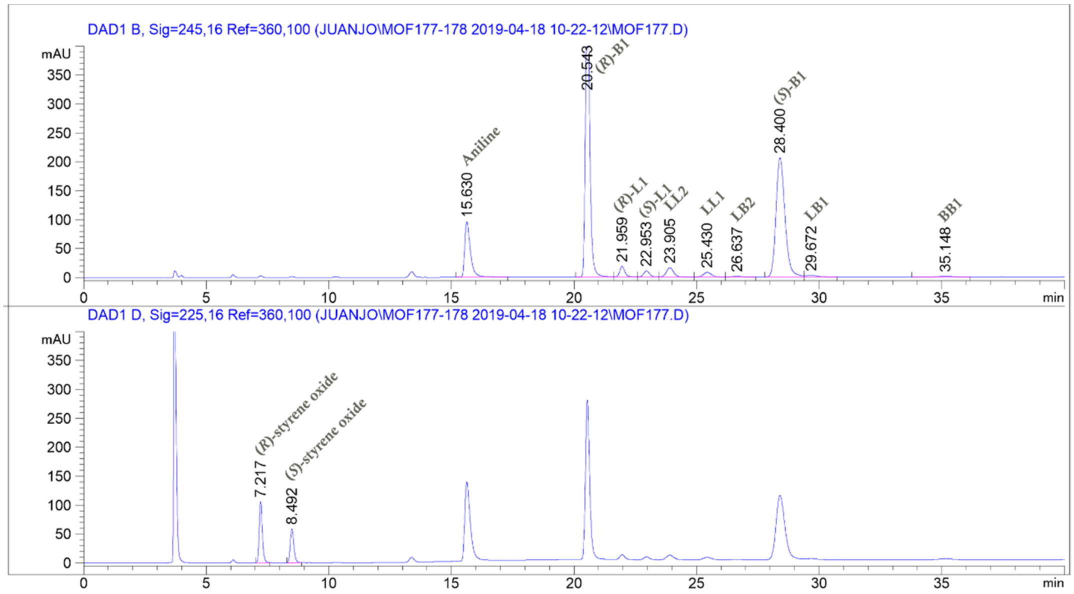

Signal 1: DAD1 B, Sig=245,16 Ref=360,100

Peak #	RetTime [min]	Type	Width [min]	Area [mAU*s]	Height [mAU]	Area %
1	15.630	VB	0.2456	1557.89001	95.84858	10.9429
2	20.543	BV	0.1858	6210.77734	511.05444	43.6256
3	21.959	VV	0.2344	279.94583	18.31028	1.9664
4	22.953	VV	0.2591	186.02582	10.89963	1.3067
5	23.905	VV	0.3252	340.22998	16.18790	2.3898
6	25.430	VV	0.3597	189.91560	8.15403	1.3340
7	26.637	VB	0.3967	43.84831	1.70117	0.3080
8	28.400	BV	0.3931	5252.01465	206.22620	36.8911
9	29.672	VB	0.5014	104.16222	3.08894	0.7317
10	35.148	BB	0.5934	71.74158	1.87934	0.5039

Signal 2: DAD1 D, Sig=225,16 Ref=360,100

Peak #	RetTime [min]	Type	Width [min]	Area [mAU*s]	Height [mAU]	Area %
1	7.217	BB	0.1357	941.10889	105.86442	61.2899
2	8.492	BB	0.1564	594.39441	58.55555	38.7101

Figure ESI21. HPLC chromatogram of a representative reaction crude mixture of *rac*-**1** and aniline (**2**) (top: 245 nm; bottom: 225 nm). Conditions: CHIRALPAK® IC, *n*-Hex/IPA 99:1 to 95:5, 1 mL/min, gradient (min, %IPA): (0, 1); (7, 1); (14, 5); (40, 5).

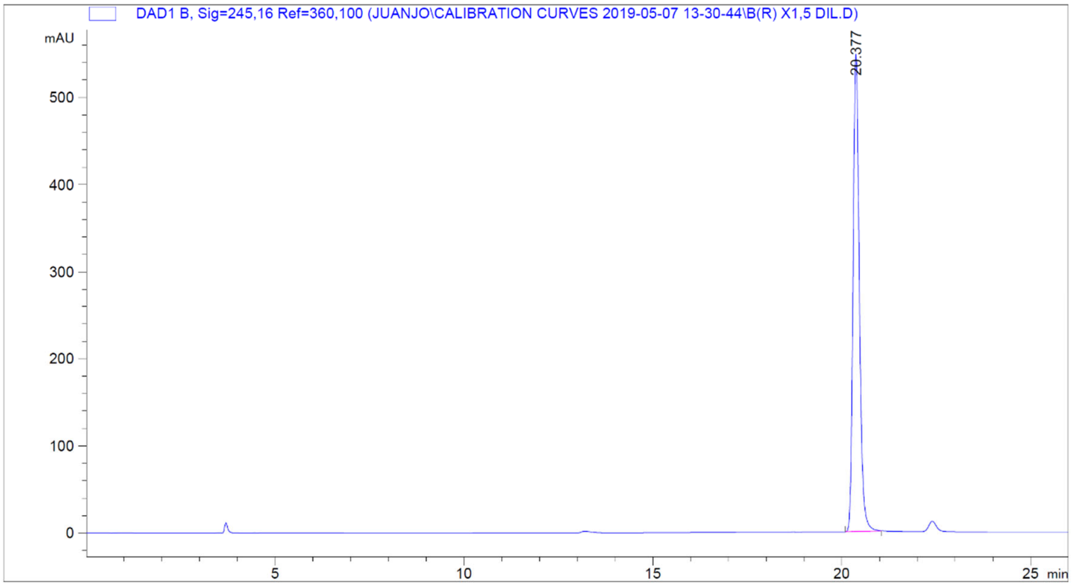

Figure ESI22. HPLC chromatogram of enantiopure (*R*)-**B1**. Conditions: CHIRALPAK® IC, *n*-Hex/IPA 99:1 to 95:5, 1 mL/min, gradient (min, %IPA): (0, 1); (7, 1); (14, 5); (40, 5).

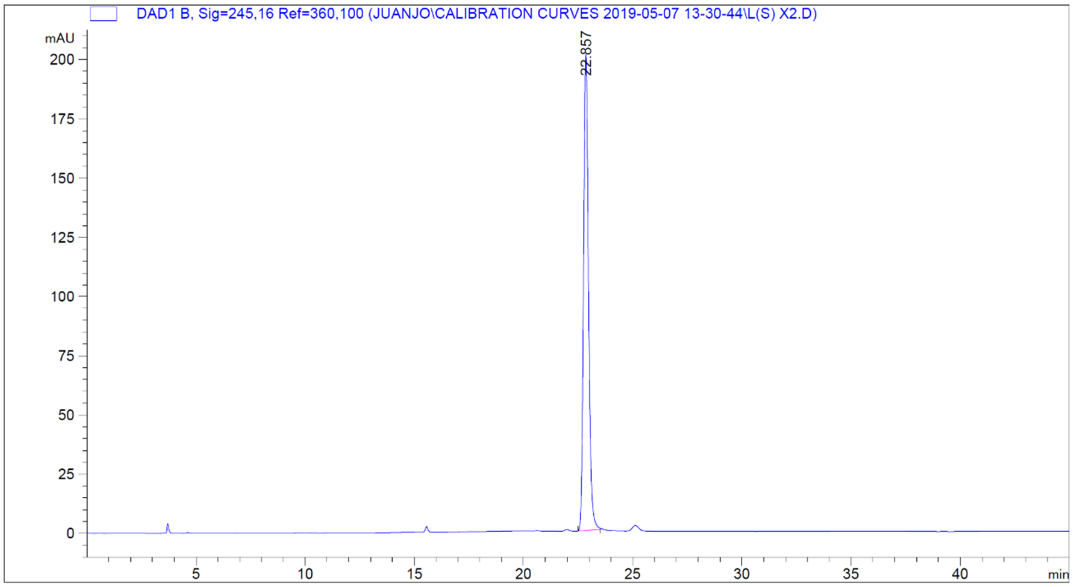

Figure ESI23. HPLC chromatogram of enantiopure (*S*)-**L1**. Conditions: CHIRALPAK® IC, *n*-Hex/IPA 99:1 to 95:5, 1 mL/min, gradient (min, %IPA): (0, 1); (7, 1); (14, 5); (40, 5).

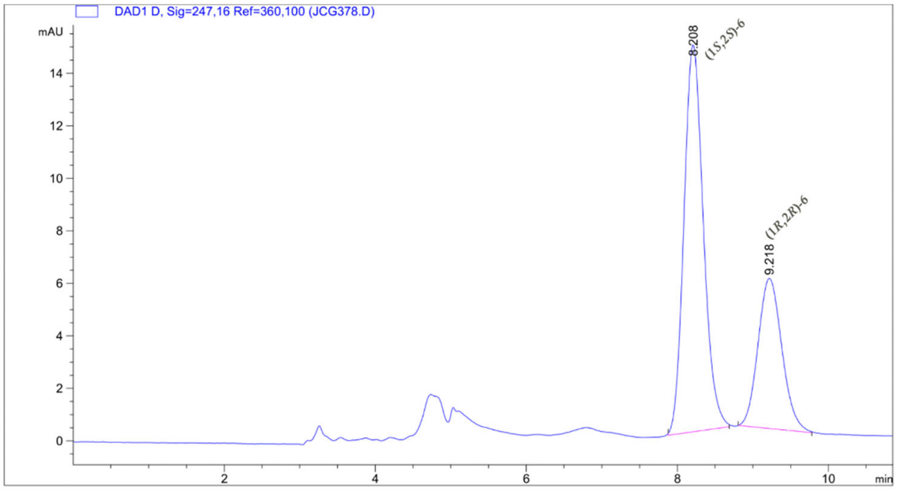

Signal 1: DAD1 D, Sig=247,16 Ref=360,100

Peak #	RetTime [min]	Type	Width [min]	Area [mAU*s]	Height [mAU]	Area %
1	8.208	BB	0.2741	262.77628	14.73032	67.7085
2	9.218	BB	0.3399	125.32330	5.71467	32.2915

Figure ESI24. HPLC chromatogram of the reaction of **5** and aniline (**2**) catalyzed by **TAMOF-1**. Conditions: CHIRALCEL® OD-H, *n*-Hex/IPA 85:15, 1 mL/min, 247 nm.[4]

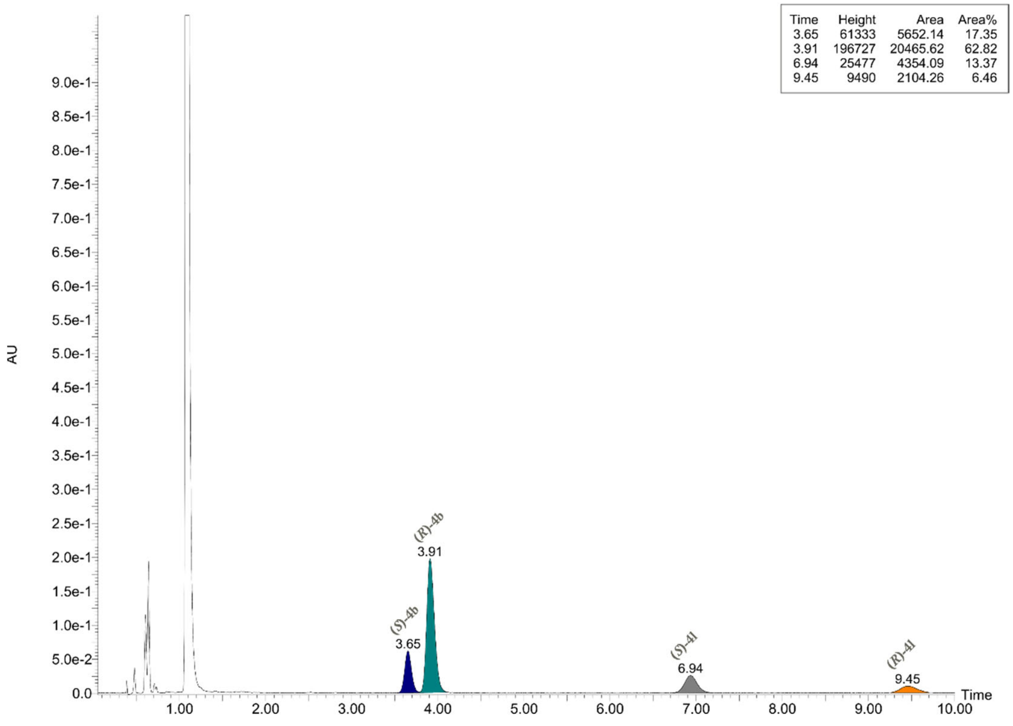

Figure ESI25. SFC chromatogram of the reaction of *rac*-**1** and *o*-anisidine (**3**) catalyzed by **TAMOF-1**. Acquity UPC2, CHIRALPAK® IA column, CO_2/MeOH 95:5, 1500 psi, 240 nm.

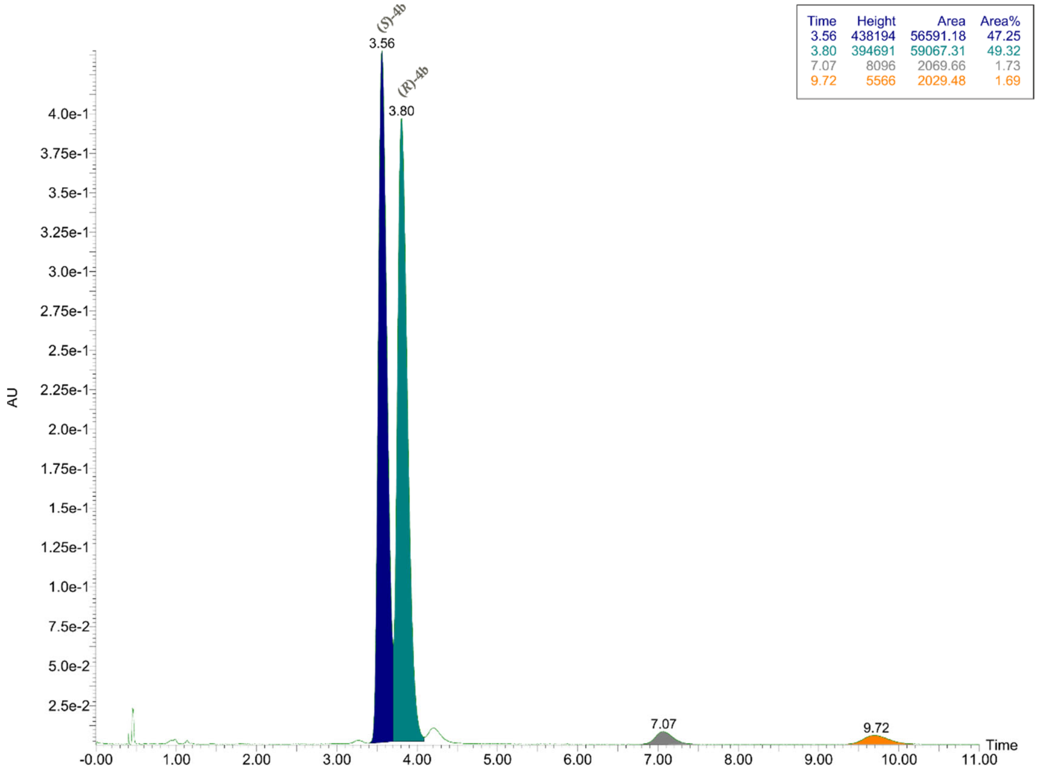

Figure ESI26. SFC chromatogram of *rac*-**4b**. Acquity UPC2, CHIRALPAK® IA column, CO_2/MeOH 95:5, 1500 psi, 240 nm.

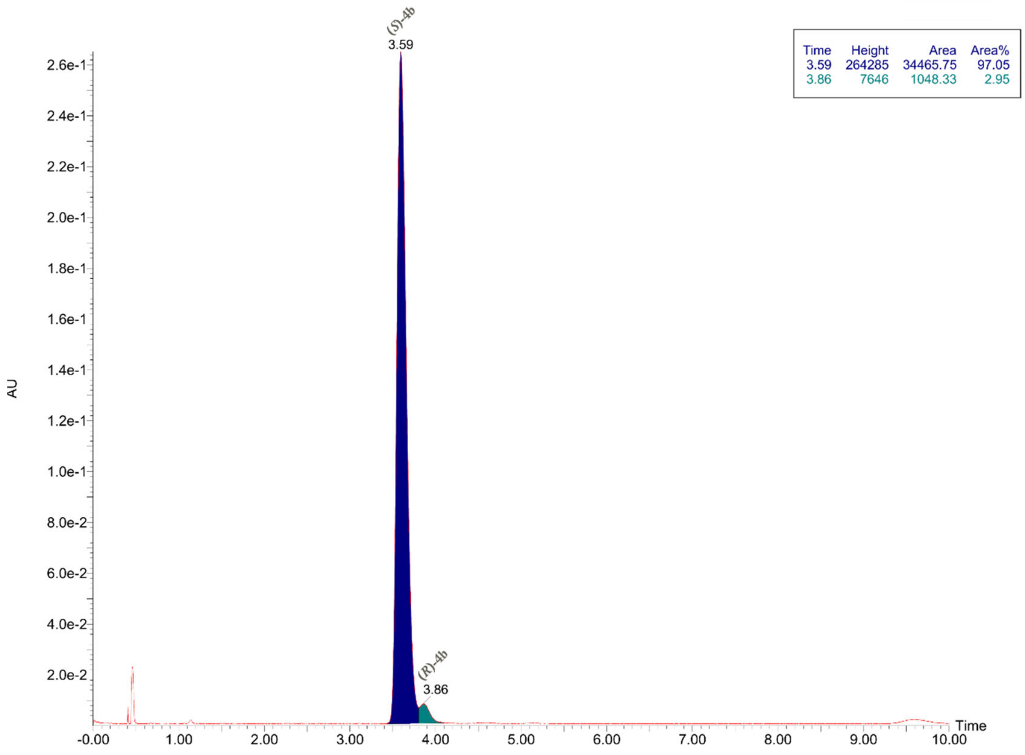

Figure ESI27. SFC chromatogram of pure (*S*)-**4b**. Acquity UPC2, CHIRALPAK® IA column, CO_2/MeOH 95:5, 1500 psi, 240 nm.

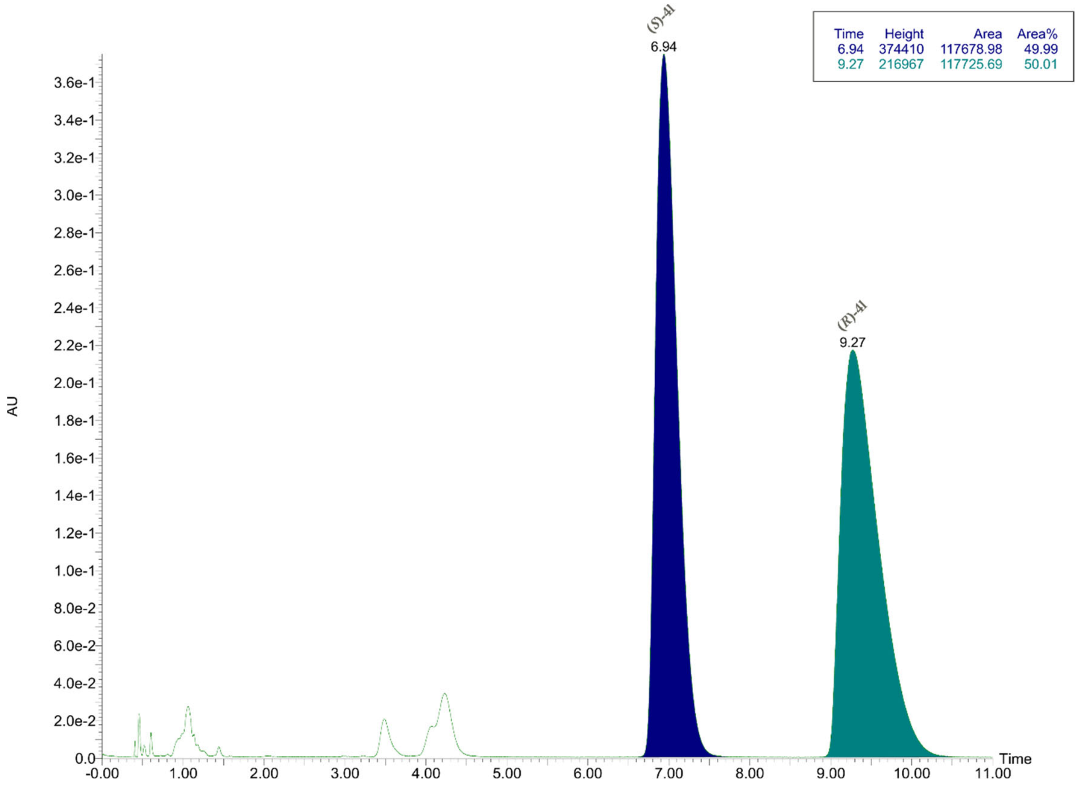

Figure ESI28. SFC chromatogram of *rac*-**4l**. Acquity UPC2, CHIRALPAK® IA column, CO_2/MeOH 95:5, 1500 psi, 240 nm.

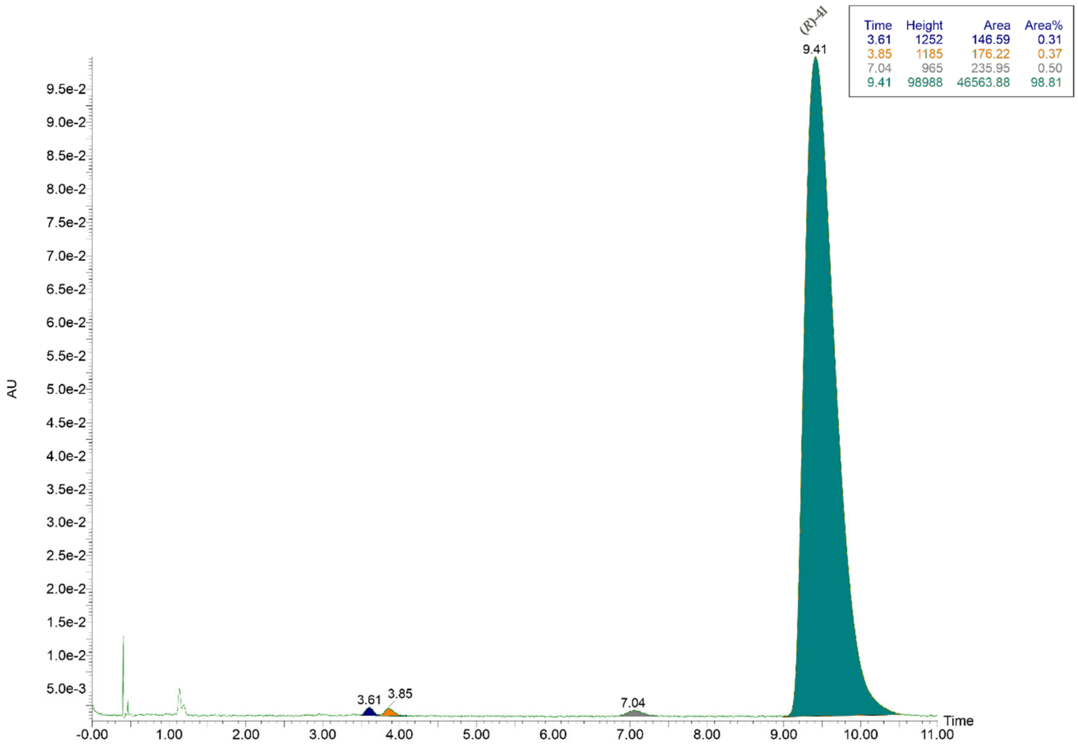

Figure ESI29. SFC chromatogram of pure (*R*)-**4l**. Acquity UPC2, CHIRALPAK® IA column, CO_2/MeOH 95:5, 1500 psi, 240 nm.

References

[1] R. K. Bartlett, I. R. Humphrey, *Journal of the Chemical Society C: Organic* **1967**, 1664.

[2] A. B. Lysenko, G. A. Senchyk, K. V. Domasevitch, M. Kobalz, H. Krautscheid, J. Cichos, M. Karbowiak, P. Neves, A. A. Valente, I. S. Gonçalves, *Inorg. Chem.* **2017**, *56*, 4380-4394.

[3] M. N. Corella-Ochoa, J. B. Tapia, H. N. Rubin, V. Lillo, J. González-Cobos, J. L. Núñez-Rico, S. R. G. Balestra, N. Almora-Barrios, M. Lledós, A. Güell-Bara, J. Cabezas-Giménez, E. C. Escudero-Adán, A. Vidal-Ferran, S. Calero, M. Reynolds, C. Martí-Gastaldo, J. R. Galán-Mascarós, *Journal of the American Chemical Society* **2019**, *141*, 14306-14316.

[4] E. Mai, C. Schneider, *Chem. Eur. J.* **2007**, *13*, 2729-2741.

[5] A. Ziyaei-Halimehjani, H. Gholami, M. R. Saidi, *Journal of the Iranian Chemical Society* **2013**, *10*, 7-11.

[6] S. S. Chimni, N. Bala, V. A. Dixit, P. V. Bharatam, *Tetrahedron* **2010**, *66*, 3042-3049.

[7] G. Xu, G. Yang, Y. Wang, P.-L. Shao, J. N. N. Yau, B. Liu, Y. Zhao, Y. Sun, X. Xie, S. Wang, Y. Zhang, L. Xia, Y. Zhao, *Angew. Chem. Int. Ed.* **2019**, *58*, 14082-14088.

[8] D. Li, J. Wang, S. Yu, S. Ye, W. Zou, H. Zhang, J. Chen, *Chem. Commun.* **2020**, *56*, 2256-2259.

[9] R. Tak, M. Kumar, T. Menapara, N. Gupta, R. I. Kureshy, N.-u. H. Khan, E. Suresh, *Adv. Synth. Catal.* **2017**, *359*, 3990-4001.

[10] Z. Xu, S. Zhu, Y. Liu, L. He, Z. Geng, Y. Zhang, *Synthesis* **2010**, 811-817.
